# Promotion of virus assembly and organization by the measles virus matrix protein

**DOI:** 10.1038/s41467-018-04058-2

**Published:** 2018-04-30

**Authors:** Zunlong Ke, Joshua D. Strauss, Cheri M. Hampton, Melinda A. Brindley, Rebecca S. Dillard, Fredrick Leon, Kristen M. Lamb, Richard K. Plemper, Elizabeth R. Wright

**Affiliations:** 1 Division of Infectious Diseases, Department of Pediatrics, Emory University School of Medicine, Children’s Healthcare of Atlanta, Atlanta, GA 30322 USA; 2 School of Biological Sciences, Georgia Institute of Technology, Atlanta, GA 30332 USA; 3 Department of Infectious Diseases, Department of Population Health and Center for Vaccines and Immunology, University of Georgia, Athens, GA 30602 USA; 4 Institute for Biomedical Sciences, Georgia State University, Atlanta, GA 30303 USA; 5 Robert P. Apkarian Integrated Electron Microscopy Core, Emory University, Atlanta, GA 30322 USA

## Abstract

Measles virus (MeV) remains a major human pathogen, but there are presently no licensed antivirals to treat MeV or other paramyxoviruses. Here, we use cryo-electron tomography (cryo-ET) to elucidate the principles governing paramyxovirus assembly in MeV-infected human cells. The three-dimensional (3D) arrangement of the MeV structural proteins including the surface glycoproteins (F and H), matrix protein (M), and the ribonucleoprotein complex (RNP) are characterized at stages of virus assembly and budding, and in released virus particles. The M protein is observed as an organized two-dimensional (2D) paracrystalline array associated with the membrane. A two-layered F–M lattice is revealed suggesting that interactions between F and M may coordinate processes essential for MeV assembly. The RNP complex remains associated with and in close proximity to the M lattice. In this model, the M lattice facilitates the well-ordered incorporation and concentration of the surface glycoproteins and the RNP at sites of virus assembly.

## Abstract (web summary)

Virus assembly is technically challenging to study. Here the authors use cryo-electron tomography of measles virus-infected human cells to determine native-state virus structure and they locate well-ordered M lattices that organize viral glycoproteins, RNP, and drive assembly.

## Introduction

Paramyxoviruses are defined as pleomorphic, enveloped, non-segmented, and negative-sense single-stranded RNA viruses^1^. Many of these viruses cause notable diseases in humans and animals. Members of the family include, amongst others, human parainfluenza viruses (HPIV 1–4), MeV, Hendra virus, Nipah virus, avian paramyxovirus 1 (APMV-1, also known as Newcastle disease virus, and will be referred as NDV thereafter), and Sendai virus^1^. Although an experimental viral polymerase inhibitor was shown to be orally efficacious against members of the morbillivirus genus that includes MeV^2^, there are limited antivirals against several members of the *Paramyxoviridae* family. Paramyxoviruses enter the host cell via viral glycoprotein attachment to host cell receptors followed by membrane fusion^3–6^. The replication process proceeds in the cell’s cytoplasm and assembly of virus particles occurs at the cell plasma membrane^4, 7^. The process is completed when the infectious virus buds from the host cell membrane or infects a neighboring cell^8, 9^. Some common mechanisms are known to regulate paramyxovirus assembly and budding^4^; however, there is limited native-state structural information of these processes in the context of an infected host cell.

MeV is a canonical member of the *Paramyxoviridae* family^1^. The MeV RNA genome encodes six structural proteins: two membrane-anchored surface glycoproteins (fusion glycoprotein (F) and attachment glycoprotein hemagglutinin (H)); the matrix protein (M); the nucleoprotein (N), which forms the ribonucleoprotein complex (RNP) that encapsidates the viral RNA genome; and the large polymerase protein (L) and the phosphoprotein (P) that form the RNA-dependent RNA polymerase (RdRp)^10, 11^. The M protein is thought to drive MeV assembly by physically recruiting the RNP and glycoproteins to the host cell’s plasma membrane^12–15^. X-ray crystallographic studies of the M protein from NDV^16^ and the related *Pneumoviridae* family including respiratory syncytial virus (RSV)^17^ and human metapneumovirus (HMPV)^18^ suggest that M proteins associate with the cell membrane by electrostatic interactions. Studies have shown that altered interaction between M and the cytoplasmic tail of H or F affects MeV viral growth^15, 19^, indicating the necessity for contacts between M and the glycoproteins during assembly. Recent structural studies of NDV by cryo-ET and X-ray crystallography demonstrated that the RNP complex is aligned with M protein arrays^16^. Furthermore, it has been suggested that actin filaments play a role in the MeV assembly and budding process by facilitating the transportation of M-RNP complexes^20, 21^.

Cryo-ET studies of purified MeV particles^22–24^ have provided the first insight into the presence and organization of the structural proteins in isolated virions. From these cryo-ET studies, no obvious glycoprotein ordering was observed^22^ even upon imaging a recombinant MeV strain (recMeV-(H-118∇41×)) that harbors a modified H protein with an extension of the helical stalk domain by 41 amino acids^23, 25^. A tomography study of HPIV3 illustrated that there is no local or long-range organization of the glycoproteins. Large ordered arrays of HN tetramers in the ‘heads down’ conformation were present on HPIV3 particles, but only in the absence of F, and F ordering was not present on any of the particles^26^. Cryo-EM and cryo-ET investigations of other paramyxoviruses and pneumoviruses have also indicated that there is no local or long-range ordering of the fusion glycoproteins^16, 27–29^. However, in a recent structural study of the fusion glycoprotein of Nipah virus, Xu et al. reported that the fusion glycoprotein is capable of forming a hexamer of pre-fusion trimers in the context of a protein crystal and by negative stain TEM^30^. The authors propose that this arrangement may stabilize the F protein in the pre-fusion state prior to cell attachment. Furthermore, this organization could enhance the coordinated triggering of the F trimers to support membrane fusion and fusion pore formation. It is possible that this pattern of organization may exist on native paramyxovirus particles; however, this coordinated structure of glycoproteins has yet to be visualized. All together, these findings have provided information about the 3D structure of intact viruses, the isolated F protein, and prospective models for the attachment and fusion process.

A longstanding question in paramyxovirus biology is: do the M proteins of the paramyxoviruses all function similarly to each other to support virus assembly and budding or are there alternative pathways, especially in the case of MeV? Recently, Liljeroos et al.^22^ proposed a MeV assembly model in which the M protein coats the RNP complex as an external helical structure, without M present at the viral membrane. In closely related NDV, Battisti et al. demonstrated that M and the RNP complex are in register with each other along the viral membrane in virions^16^. Many studies have reported negative stain TEM or cryo-EM structures of purified viruses, in which the methods of virus production and purification may alter the virus particles sufficiently to impact the interpretation of the data. Cryo-EM investigations of pleomorphic viruses by whole-cell tomography methods, where the viruses are preserved on the grid during the process of assembly and just after release from the host cell, have improved our understanding of human immunodeficiency virus type-1 (HIV-1) assembly and particle restriction^31, 32^, Marburg virus budding^33^, as well as the incorporation and order of RSV glycoproteins on intact virions^34^. Broader application of this method to paramyxoviruses and other enveloped viruses will prove useful for defining the mechanisms that regulate virus replication.

To date, we enjoy a relatively rich landscape of high-resolution structures of purified individual components of MeV, including H^35, 36^, RNP^10^, and structural homologs of M and F^16, 37^, but there is limited native-state 3D information that defines the processes of MeV assembly and budding. To explore the structural mechanisms of MeV assembly and budding, we used whole-cell cryo-ET to directly visualize MeV-infected human cells. Here, we report that the M protein is present as 2D arrays with discrete C4 symmetrical unit cells on the inner leaflet of the plasma membrane at the MeV assembly and budding sites, and in released viral particles. In addition, M makes coordinated contacts with the surface glycoproteins and the RNP complex that regulated the overall architecture of MeV particles. Sub-volume averaging reveals ordering of the F glycoproteins above the M protein arrays that account for a two-layered lattice (F–M lattice). These findings support a model in which M is the driving force for MeV assembly via the formation of a well-ordered M lattice on the cell plasma membrane through which the glycoproteins and the RNP complex are coordinated.

## Results

### Cryo-ET structures of MeV assembly and released particles

**Fig. 1:**
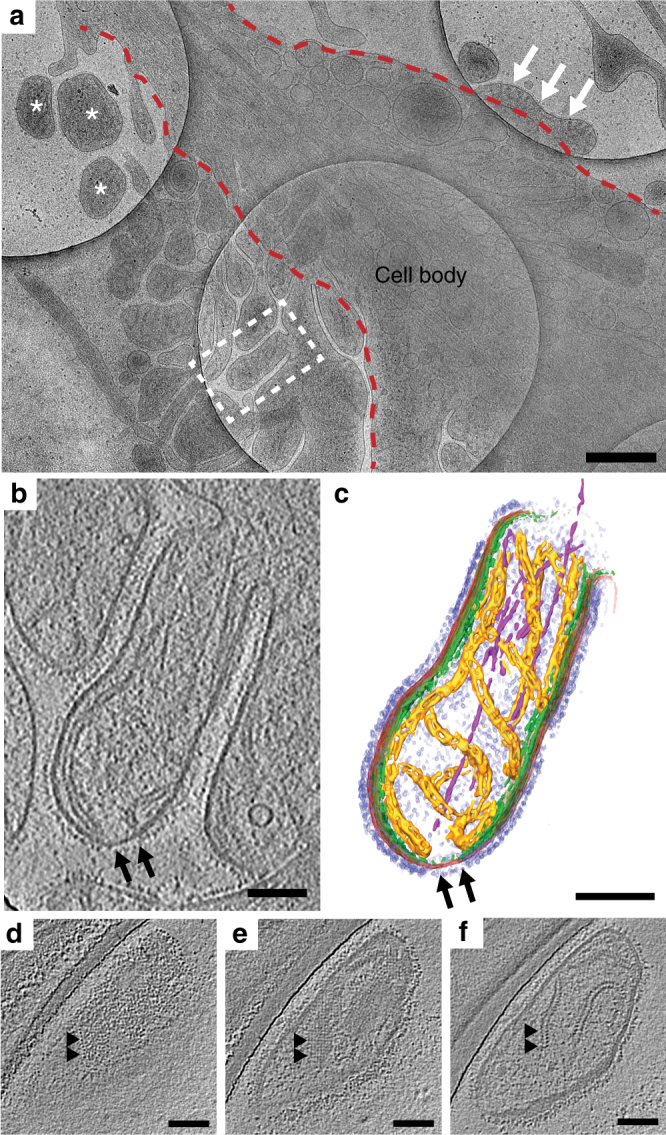
Cryo-ET of MeV-infected HeLa cell. **a** An intermediate magnification image montage of an Edm MeV-infected HeLa cell. Site of MeV assembly is indicated by dashed white box and is the location where the tilt series was acquired. Asterisks indicate released MeV particles, white arrows indicate MeV assembly site, and red dashed lines indicate the cell membrane. **b** Tomographic slice (1.18 nm thick) of Edm MeV assembly site. Black arrows indicate areas where M is absent on the viral membrane (**b**, **c**). **c** 3D segmentation view of the cryo-ET data (**b**). Glycoproteins (purple), viral membrane (red), M (green), RNP (gold), and actin filaments (magenta) at the assembly site. **d**–**f** Sequential tomographic slices (1.18 nm thick) of a released virus particle (not shown in **a**) showing glycoprotein layer (**d**), M layer (**e**) and RNP layer (**f**). Arrowheads indicate the same regions at different *Z* heights. Scale bars are 500 nm (**a**) and 100 nm (**b**–**f**)

Our objective was to determine the arrangement of the MeV surface glycoproteins (F and H), M, and the RNP complex during virus assembly, budding, and particle release using whole-cell cryo-ET. Sites of MeV assembly were resolved by 2D cryo-TEM imaging of MeV-infected HeLa and MRC-5 cells (Fig. 1a, Supplementary Tables 1-2, Supplementary Movies 1-2). In these regions, the exterior of cellular membranes was decorated with the surface glycoproteins and the M protein lined the interior face of the membrane, with the RNP associating only with the M-lined regions (Fig. 1b, c, Supplementary Figure 1, Supplementary Movies 1-3). In the cryo-ET data, we resolved in 3D the glycoproteins, M protein, and the RNP complex at macromolecular resolution (2–5 nm) (Fig. 1b–f, Supplementary Figures 1-2, Supplementary Movies 1-4, Supplementary Table 3). In order to fully assess the presence and placement of the two surface glycoproteins, F and H, we infected cells with a previously characterized recombinant MeV (recMeV) strain in which the H protein has an extended stalk domain^23, 25^. We refer to the recMeV strain as recMeV-(H-118∇41×) and the standard Edmonston strain as Edm. In our cryo-ET data, the F and H proteins were interspersed and rarely partitioned into separate clusters consisting of only F or H. The ultrastructure of the Edm and recMeV-(H-118∇41×) assembly sites and released particles were indistinguishable from one another in terms of gross morphology and in the physical arrangement of the MeV structural proteins (Fig. 1, Supplementary Figures 1-2, Supplementary Movies 1–4).

The glycoproteins and RNP were associated with areas of the membrane that contained M protein, as seen in linear density profiles (Supplementary Figures 1-2). In Edm MeV, density peaks corresponding to the surface glycoproteins were 11 nm above the membrane, the M protein peak was 6 nm below the membrane, and the two RNP peaks were located 17 and ~29 nm below the membrane (Supplementary Figure 1). In comparison, analysis of recMeV-(H-118∇41×) revealed that the two surface glycoproteins were present at different heights above the membrane, with F located ~8 nm above the membrane and H at an extended height of ~19 nm above the membrane (Fig. 1b–c, Supplementary Figures 1-2). The M protein was observed on the cytoplasmic face of the plasma membrane and on the inner face of the viral membrane (Fig. 1, Supplementary Figures 1-2). In both viruses, the glycoproteins, F and H, were preferentially located on M-lined areas of the membrane. Areas of the viral and cellular membrane that lacked M protein were decorated with significantly fewer glycoproteins (Fig. 1b, c, Supplementary Figure 3).

### In situ structure of MeV M protein 2D array

**Fig. 2:**
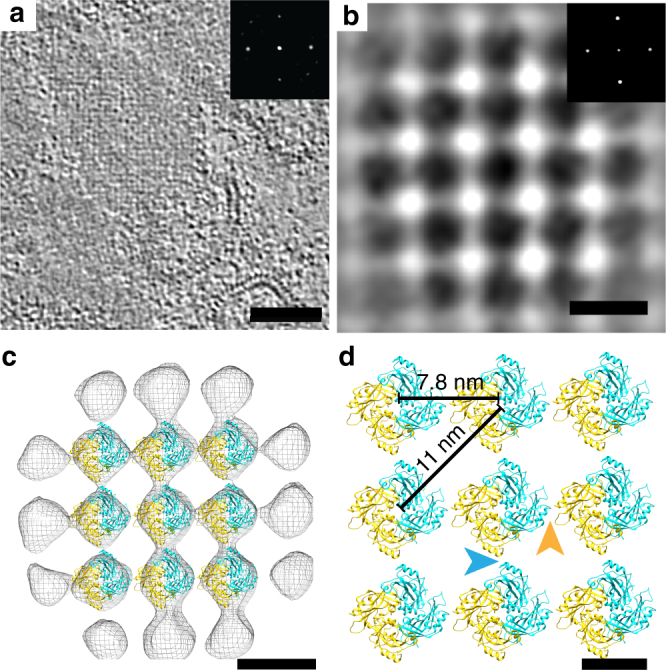
MeV M protein forms a 2D paracrystalline array. **a** Tomographic slice showing a representative M array from Edm MeV-infected HeLa cells. Inset is the power spectrum indicating a four-fold symmetric structure with 7.8 nm spacing between subunits. **b** Sub-volume average of the M protein array indicates an improved SNR structure with regular spacing. Black is density. **c** Model fitting of the NDV M dimer into the averaged MeV M array density. Cyan and gold represent each monomer of the NDV M dimer. **d** The enlarged view of (**c**), with blue and gold arrowheads indicate the two dimer interfaces. Scale bars are 100 nm (**a**), 10 nm (**b**, **c**), and 5 nm (**d**)

To gain further insight into the role of the MeV M protein in driving virus assembly and promoting virus structural organization^12–15^, we examined the MeV M protein structure from MeV-infected cells and released MeV particles. During virus assembly and in released MeV particles, we visualized a layer of density lying underneath the viral membrane, and we assigned this density to the M protein layer (Fig. 1 and Supplementary Figures 1-2)^22^. Further inspection of this layer showed the presence of higher-ordered M protein assemblies (Fig. 2). Power spectra from regions exhibiting well-ordered patches of the M protein illustrated that the 2D arrays maintained C4 symmetry with subunit spacing of 7.8 nm (Fig. 2a).

To better resolve the structure of the subunits forming the M layer, we used sub-volume averaging. Because the MeV assembly sites and particles were pleomorphic with respect to membrane curvature, distribution of M lattices, and other viral factors, we limited the sub-volume analyses to flat regions where the M lattice was clearly resolved in the tomographic slices. Sub-volume averages were generated from individual tomograms of both the Edm and the recMeV-(H-118∇41×) viruses, and both indicated that the M lattice 2D arrays had four-fold symmetry (C4). No symmetry was imposed during the alignment and averaging process. Power spectra of the averaged structure demonstrated that the spacing between the nearest M subunits was ~7.8 nm (Fig. 2), which was consistent with the raw tomographic data. In order to validate the spacing and subunit arrangement, the NDV M dimer^16^, a structural homolog of MeV M, was modeled into the averaged 3D volume. The crystal structure density corresponded to the averaged EM density and the MeV M subunit contact points in the hypothetical model revealed plausible similarities with that of NDV M protein (Fig. 2c, d).

### F glycoprotein forms a 2D lattice with four-fold symmetry

**Fig. 3:**
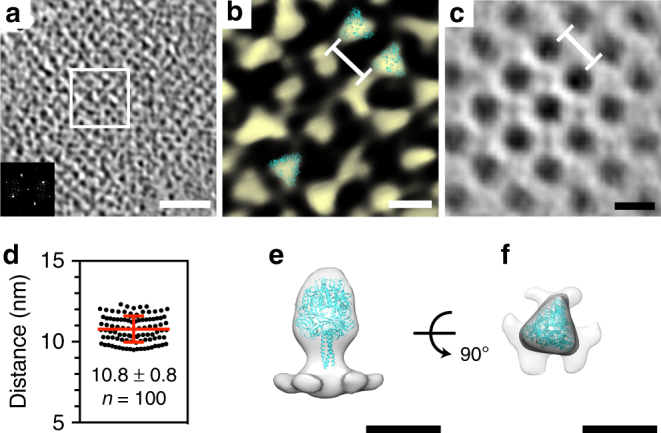
MeV F glycoprotein forms a 2D paracrystalline lattice with four-fold symmetry. **a** Tomographic slice showing the F glycoprotein layer from recMeV-(H-118∇41×)-infected HeLa cells at an assembly site. The inset is the power spectrum of the F layer, which indicates four-fold symmetry with subunit spacing of ~11 nm. Black is density. **b** The enlarged region (**a**) (white box) with an isosurface rendering showing the F ordering. Three F trimer densities were fitted using PIV5 pre-fusion F glycoprotein structure (PDB ID: 2B9B). **c** Sub-volume averaged structure of the F layer showing spacing with four-fold symmetry. Black is density. **d** Measurements between two nearest neighboring F glycoproteins (white line in **b**) indicate an average distance of ~11 nm, consistent with the power spectrum analysis and the sub-volume averaged structure (white line in **c**). The error bar represents the standard deviation (s.d.) of 100 measurements (graph data points). **e**, **f** Sub-volume average of F glycoprotein trimer indicates F is in the pre-fusion conformational state, validated by model fitting of PIV5 prefusion F structure (PDB ID: 2B9B). Scale bars are 50 nm (**a**) and 10 nm (**b**, **c**, **e**, **f**)

To analyze the ordering of the glycoproteins, we used recMeV-(H-118∇41×) as our study target because the two glycoproteins were distinguishable due to the separation in height of the head domains (Fig. 3, Supplementary Figures 1-2, Supplementary Movie 4)^23, 25^. In most areas within the assembly sites or on released virus particles, individual glycoproteins could be appreciated, but no specific order of the glycoproteins relative to each other emerged. At some assembly sites, we identified regions with high order at the level of the F layer (Fig. 3). Power spectra generated from these regions showed a clear four-fold symmetry with an F subunit spacing of ~11 nm (Fig. 3a, inset). This distance (11 nm) corresponds to the diagonal of a square with sides of 7.8 nm, which is consistent with the measured M subunit spacing. Sub-volume averaging of the F layer demonstrated that the F glycoprotein could assemble as an extended lattice with four-fold symmetry and subunit spacing of ~11 nm (Fig. 3c). The organization identified in both the raw data and the sub-volume averages was validated by individual measurements of glycoprotein arrays along the plane of the membrane and revealed a spacing of 10.8 nm (with a standard deviation (s.d.) of ± 0.8 nm; *n* = 100) (Fig. 3d). We then docked a MeV pre-fusion F homolog (PIV5 pre-fusion F, PDB ID: 2B9B)^37^ into the raw EM densities of the F glycoproteins (Fig. 3b). The PIV5 F structure coordinated well with the trimeric shape of the MeV pre-fusion F density that was visible in the raw tomogram. We further confirmed that the trimeric densities were associated with pre-fusion MeV F by generating sub-volume averages from an isolated patch of F (Fig. 3e–f) and regions intermixed with F and H (Supplementary Figure 4). In both cases, the sub-volume averages provided confirmation that the densities were MeV F. Interestingly, the four-fold symmetry lattice of MeV F resolved by whole-cell cryo-ET is distinct from the more irregular arrangements observed by cryo-ET of purified virus particle preparations of Sendai virus^27^, RSV^28, 29^, and HPIV3^26^ as well as the well-ordered, highly packed hexameric structure of the Nipah virus fusion glycoprotein determined by X-ray crystallographic means^30^.

### The organization of a double-layered F–M lattice

**Fig. 4:**
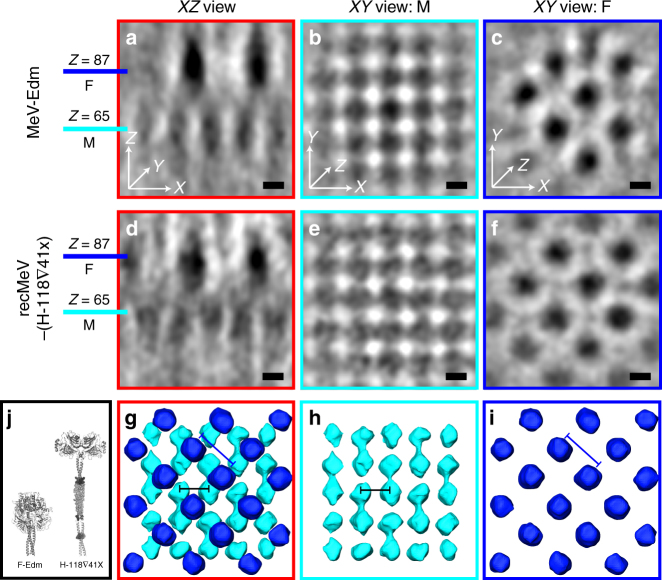
Sub-volume average of the MeV F–M lattice layers. **a**-**f** Tomographic slices of the ordered F–M lattice. Pixel size is 5.88 Å at binning 2. Central slices at *Z* = 65 (black, M) and *Z* = 87 (blue, F) are shown for Edm (**a**–**c**) and recMeV-(H-118∇41×) (**d-f**). **g**-**i** Segmentation view of recMeV-(H-118∇41×) F–M lattice demonstrates the densities corresponding to M (**h**), F (**i**) and the F–M lattice overlay (**g**, F on top of M). Subunit distance between M (black line) is 11 nm and F (blue line) is 7.8 nm. Black is density in **a**–**f**. **j** Graphic representation of F and H in recMeV-(H-118∇41×) virus strain. Note the height differences in F and H in this virus strain. Black is density. Scale bars are 5 nm

In order to assess whether M and F can assemble into a complex that maintains two overlaid lattices (F–M lattice), we carried out sub-volume averaging on larger volumes that contained both M and F. After iterative alignment and refinement, a structure with a two-layer lattice complex emerged: the M lattice under the membrane with four-fold symmetry and the F lattice above (Fig. 4, Supplementary Movies 5-7). The M lattice from the two-layer F–M lattice structure (Fig. 4) was indistinguishable from the M-only lattice (Fig. 2); the spacing and lateral coverage were comparable, with ~7.8 nm spacing between the subunits. The F lattice from F–M lattice complex has ~11 nm subunit spacing, which corresponds to the diagonal of a square with sides of 7.8 nm, and was equivalent to the F-only lattice (Fig. 3a–c). In addition, the central density of the individual units of the F lattice was ~12 nm above the densities associated with the M lattice, which was consistent with the linear density profiles (Fig. 4, Supplementary Figures 1-2). We noted that the two lattices were interspersed in a unique way that could be best appreciated when the sub-volume average was viewed as an *XY* 2D-projection. First, each F subunit was surrounded by four M densities and the distance from each M to the center F was ~5.5 nm (Figure 4g–i, Supplementary Movies 5-6). Second, the F–M lattice unit cells maintained an alternating pattern of coordination where an empty “glycoprotein” position was interspersed in the lattice. This location could be attributed to the location of the H glycoprotein, as proposed by Battisti et al. for the glycoproteins on NDV particles^16^. The initial model (see Methods section) suggests an ordering of the F layer with a spacing of 7.8 nm (Supplementary Figure 5). This initial model was generated assuming the glycoprotein has the same periodicity as the M layer, as done in the case of NDV^16^. Assuming the same periodicity between the F layer and M layer would result in an initial model with 7.8 nm spacing (Supplementary Figure 6). However, a closer inspection and analysis of the F glycoprotein layer revealed that the spacing is 11 nm (Fig. 3). We examined the organization of the H layer in both the Edm and recMeV-(H-118∇41×) viruses by extending the calculated region of the sub-volume. However, consistent spacing within the H layer was not revealed (Supplementary Figures 5, 7). To further evaluate the structural complexity of the H layer, we generated power spectra, sub-volume averages, and variance maps of the H layer with the recMeV-(H-118∇41×) data (Supplementary Figure 5). Power spectra and sub-volume averages of the MeV H layer did not show a periodic spacing of H at either 7.8 or 11 nm (Supplementary Figure 7). Variance maps indicate high variance localized on the H layer, suggesting flexibility and disorder of the H glycoproteins on MeV particles (Supplementary Figure 5). Together, these data revealed substantial structural heterogeneity in the H layer, which is consistent with other results that indicate the paramyxovirus attachment proteins are structurally flexible due to conformational states and glycosylation levels^38, 39^.

### M lattice has a tight association with the RNP complex

**Fig. 5:**
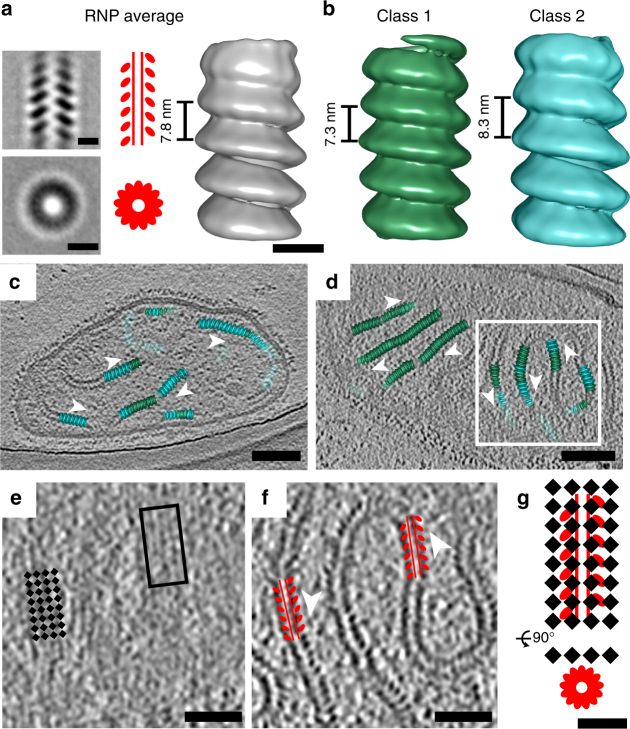
MeV RNP average and its spatial organization with M lattice. **a** Global average of MeV RNP with central slice side and top views (left), cartoon representation (middle), and isosurface rendering (right). **b** Classification reveals two classes with different helical pitches. **c**, **d** RNP class average distribution in the raw tomograms. White arrowheads indicate the directionality of the RNP. **e**, **f** The enlarged view of the white box in (**d**) showing tomographic slices of MeV at M layer (**e**) and RNP layer (**f**). Black squares indicate M lattice; red helices indicate the RNP helices. Note the directionality of M lattice is in register with RNP directionality. **g** Schematic model of M and RNP interaction and 3D organization. Black is density. Scale bars are 10 nm (**a**, **b**), 100 nm (**c**, **d**), 50 nm (**e** and **f**), and 20 nm (**g**)

In the raw data, we resolved the RNP complex directly beneath the M layer at sites of assembly and in released virus particles (Fig. 1, Supplementary Movies 3, 8). We, therefore, sought to determine whether there were coordinated associations between M and the RNP complex. First, we observed that the directionality of the extended M lattice matched the orientation of the RNP helix (Fig. 5e–g, Supplementary Movie 3, 8). Specifically, the ordered M lattice was in register with the path of the RNP complex at the sites of assembly and in the released virus particles (Fig. 5c–g). To further examine the helical RNP structure, we produced sub-volume averages of the RNP complex. The detailed procedures can be found in the Methods. The global averaged structure maintained a left-handed helix^10, 22^ with a helical pitch of 7.8 nm (Fig. 5a). Within the global average, the densities associated with the averaged structure towards the central segment of the helix were stronger while the top and the bottom segments of the RNP helix were weaker (Fig. 5a, Supplementary Figure 8). To determine if the reduced densities within the map represented variability in the RNP structure and the presence of sub-classes within the global average, we carried out principle component analysis (PCA) and K-means clustering^40^. From this, we identified two global classes of the RNP with different helical pitches, yet the ~19 nm diameter of the helices remained the same (Fig. 5b, Supplementary Figure 8). One helix class was more compact with a pitch of 7.3 nm; the other was looser with a pitch of 8.3 nm. We then placed the two helix classes into the raw tomograms and found that the RNP is quite variable even along a single continuous RNP complex, accounting for the variation in helical pitch (Fig. 5c, d, Supplementary Movie 8).

Previously, Liljeroos et al.^22^ proposed an assembly model for MeV in which the M protein coats the RNP complex as an external helical structure. However, this data was derived from preparations of purified MeV particles. These particles could have been maturation or structural intermediates, defective interfering particles, or structurally compromised due to the purification process. Our results demonstrated that the M layer formed distinct 2D lattice arrays along membranes where the surface glycoproteins and RNP complex co-organized (Figs. 1, 2), in agreement with the assembly model of closely related NDV^16^. In rare circumstances, in data from released virus particles, we observed M-coated RNP complexes that were detached from the viral membrane (Supplementary Figure 9). However, analyses of the surface glycoproteins demonstrated that they were in the extended, triggered, post-fusion conformational state (Supplementary Figure 9).

MeV is known to be polyploid and viruses are capable of incorporating more than one genome copy without impacting overall virus infectivity^41, 42^. Therefore, we measured the length of the RNP complexes^28^ to assess whether the calculated genome length within the released particles could correspond to a full-length RNP, which is ~1.3 µm^41, 42^ (Supplementary Figures 9-10, Supplementary Movies 9-10). Our calculations revealed that MeV particles contain either a truncated genome (<1.3 µm RNP length), a single genomic copy (1.3 µm RNP length), or multiple copies of the genome (>1.3 µm RNP length), as shown in Supplementary Figures 9-10. Viral particles with a truncated genome, based on the RNP length, are presumed non-viable.

In order to assess the relationship between M, the RNP, and virus infectivity, we carried out heat-treatment experiments on MeV-infected cells. It has been shown that heat-treatment (60 °C) triggers the transition of MeV prefusion F to postfusion F[6]. Heat-treatment experiments showed a drastic decrease in the viral titer within 5 min (Supplementary Figure 11). Cryo-ET indicates that when MeV-infected cells are heat-treated, the majority (85%) of the viral membranes are devoid of the M layer. When heated for 30 min, M detaches from the membrane, and the viral membranes rupture (Supplementary Figure 11). Only one viral particle of the heat-treated sample (27 particles investigated) had an M-coated RNP. In addition, that the MeV titer decreases over time^43^ suggests that there is a correlation with the structural re-organization of MeV particles (and possibly MeV M) over the same time frame.

## Discussion

The M protein is known to drive MeV assembly and budding^4, 44^ by interacting with the surface glycoproteins^12, 14^ and the RNP complex^13^ and acts to concentrate them on the host cell’s plasma membrane. Our analysis of the structural organization of MeV among the 57 tomograms and 89 assembly/budding events examined (Supplementary Table 1) revealed that the presence of the surface glycoproteins and the RNP complex are highly correlated with the presence of the M protein (Figs. 1, 5, Supplementary Figures 1, 3, Supplementary Movies 1-4), which agrees with previous cryo-ET studies of purified paramyxoviruses, such as Sendai virus^27^, NDV^16^ and the pneumovirus RSV^28, 29^.

A recent study concluded that the M protein does not attach to the viral membrane but rather forms a helical tube and tightly associates with the RNP helical structure (M-coated RNP)^22^. Our whole-cell cryo-ET data suggests that the M protein forms a 2D paracrystalline array under the viral membrane (Fig. 2), which is similar to the M organization in NDV, a closely related paramyxovirus^16^. Recently, the crystal structure of an orthomyxovirus (infectious salmon anemia virus) matrix protein was determined. The 2D lattice was formed by matrix monomers, which may highlight mechanisms that underlie matrix protein self-polymerization, membrane association, and matrix protein-RNP interactions^45^.

Furthermore, the MeV F glycoprotein formed a 2D lattice with a subunit spacing of ~11 nm (Fig. 3) that created a coordinated two-layered lattice with M and may be considered a co-assembly complex (Fig. 4, Supplementary Figure 5, Supplementary Movies 5-6). As far as we know, this is the first direct evidence showing that the MeV F and M interaction is spatially co-organized. It provides structural evidence to support a molecular mechanism in which M is the driver of virus assembly and interacts with the F glycoprotein.

Two forms of the RNP complex were reported previously, the M-coated RNP (rigid, ~30 nm in diameter)^22, 46, 47^ and uncoated RNP (flexible, ~20 nm in diameter)^10, 22, 48, 49^. In our study, the RNP complex is predominantly uncoated in released virus particles, and exclusively uncoated at the sites of assembly (Figs. 1, 5, Supplementary Figure 1, Supplementary Movies 1-4). Sub-volume averaging of the uncoated RNP complex revealed two global classes of RNP at both the assembly sites and within the released particles (Fig. 5, Supplementary Figure 8). This structural plasticity in vivo agrees with the in vitro study in that the RNP exhibits structural variation within one helical fiber^48^, indicating that this conformational flexibility is independent of other viral or host factors. It is unclear why MeV evolved to have such structural plasticity for the RNP^48^. We calculated that the averaged RNP helical pitch (7.8 nm) matches the M lattice subunit spacing (7.8 nm), which suggests that the association between the M lattice and the RNP complex is additional structural evidence for M governing the assembly process. Furthermore, the flexibility in the structure of the uncoated RNP complex is likely coupled with the pleomorphic nature of the virus particle and the requirement for the RNP to bend and mold along the trajectory of the M lattice and membrane. In rare cases a rigid M-coated RNP complex was observed in the released MeV particles (Supplementary Figure 9). It is possible that the RNP complex undergoes a structural and functional transition, either coordinated with virus maturation or with damage. It is also possible that virus particles with an M-coated RNP are non-infectious. Titration of the heat-treated samples indicates that the particles are non-infectious after incubating at 60 °C for 10 min. Cryo-ET data of the heated specimens showed that a disordered material began to aggregate inside the viral particles, which was also correlated with the detachment and loss of M from the viral membrane (Supplementary Figure 11). In these experiments, we did not observe a positive association between heat-treatment and the frequency of the M-coated RNP. Our results do lean towards a positive correlation between the presence of M along the viral membrane and higher viral titer values, which indicates a larger population of infectious particles. However, within the populations examined, no apparent relationship existed between the M-coating of the RNP and the infectious nature of the virus particles. Future studies could determine whether the RNP compresses to dimensions associated with a lower helical pitch of ~5–6 nm^48^ due to a transition state much later than the initial processes of virus assembly and release in which the RNP maintains an expanded helical pitch of ~7–8 nm (Fig. 5, Supplementary Figure 8). In addition, the mechanisms of how MeV M protein detaches from the viral membrane and coats the RNP complex as well as the biological functions of the M-coated RNP in MeV particles are still unknown and require further investigations.

**Fig. 6:**
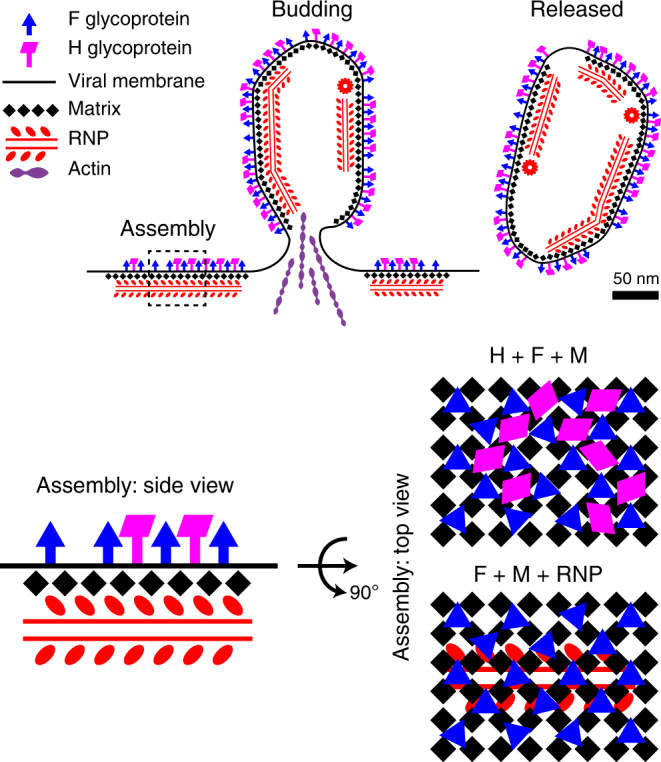
MeV assembly model. At the assembly site, viral components, including glycoproteins F and H, matrix protein (M), and RNP complexes, accumulate at the plasma membrane to initiate the assembly process. Actin filaments are potentially involved during the process. At the assembly site, M is the driving force which orchestrates the whole process by interacting with F and RNPs in a timely and spatially organized manner: M forms a 2D lattice under the envelope, F forms another 2D lattice interspersed above the M lattice, and RNP complexes lie under the M lattice with some degree of flexibility. H is incorporated at sites of assembly but does not appear with high spatial ordering. Once assembled, viral particles undergo an unknown scission event and bud off the plasma membrane. After release, the viral particles are free to infect the next cell

Based on cryo-ET studies of purified MeV, researchers proposed a model in which the M protein first coats the helical RNP in the cytoplasm of infected cells; the M-RNP complex is then co-transported to sites of assembly on the plasma membrane^22^. Whole-cell cryo-ET of MeV virus assembly provided direct evidence for us to propose an alternative assembly model (Fig. 6, Supplementary Movie 7). In this model, the M protein coalesces along the host cell membrane where it concentrates the surface glycoproteins and the RNP by direct protein–protein interactions. Budding and release are driven by membrane remodeling mediated by the M protein 2D lattice. After budding, the M protein remains attached to the viral membrane and restricts lateral movement of the glycoproteins. This study suggests that the oligomerization of the MeV M protein into ordered arrays regulates the spatial arrangement of the surface glycoproteins and the RNP complex, and that this is a common assembly mechanism shared among members of the paramyxovirus family.

## Methods

### Cryo-grid preparation

Details of sample preparation were described previously^50^. HeLa (ATCC CCL-2), MRC-5 (CCL-171), and Vero (CCL-81) cells were maintained in DMEM medium supplemented with 10% fetal bovine serum (FBS), 1 µg ml^−1^ penicillin, streptomycin, and amphotericin B (PSA) antibiotics. Cells were maintained at 37 °C with 5% CO_2_. 50,000–100,000 cells were seeded on gold R 2/1 Quantifoil TEM grids (Quantifoil) in MatTek dishes (MatTek Corp) 16–24 h prior to infection. Cells were inoculated with MeV strains (Edm or recMeV-(H-118∇41×), provided by Professor Richard Plemper, Georgia State University) at a multiplicity of infection (MOI) of 1, 2, 6, or 10. After 24–48 h incubation, the MeV-infected cells on the grids were plunge frozen in liquid ethane using the Gatan Cryoplunge 3 (Gatan, Pleasanton, CA) after applying 4 µl of BSA-treated 10 nm gold fiducials (EMS)^50^ directly onto the grid. Cryo-grids were stored in liquid nitrogen until imaged.

### MeV thermal stability assay

For the heat treatment experiment, HeLa cells were seeded onto gold R 2/1 Quantifoil grids in MatTek dishes and infected at a MOI of 2 with MeV Edm strain. Twenty-four hours post infection, the MatTek dishes containing the infected cells were incubated at 60 °C for the indicated time points^6^. Immediately after heat treatment, the grids were plunge frozen as described above. Meanwhile, the remaining infected cells from the same MatTek dish (where the grids were cultured) were scraped, harvested, and subjected to TCID_50_ titration.

Virus titration was performed using the endpoint method and the virus titer was expressed as TCID_50_ per ml (TCID_50_/ml) calculated with the Spearman-Karber method^51, 52^. Briefly, the virus sample was serially diluted from 10^−1^ to 10^−6^. Fifty microliter of the diluted virus was added to the confluent Vero cells in 96-well plates. Eight replicates were performed at each dilution for each sample. The infected cells were incubated for 4 days before determining the titration based on the cytopathogenic effect. Three independent experiments were performed to show the effect of heat treatment on virus titer and structural organization.

### Data collection and 3D reconstruction and image processing

Data collection and image processing procedures were previously outlined^50^ and parameters used for data collection are presented in the supplementary materials (Supplementary Table 2). Cryo-ET data collection was carried out using a JEOL JEM-2200FS 200 kV FEG-TEM (JEOL Ltd., Tokyo, Japan) with an in-column Omega energy filter (slit width 20 eV). Polygon montages were collected at 10,000× nominal magnification using a US4000 4k x 4k camera (Gatan, Pleasanton, CA). Tilt series images were collected on a DE-20 ~5k x 4k direct electron detector (Direct Electron LP, San Diego, CA) in movie mode. Images were acquired at magnifications resulting in effective pixel sizes of 2.94 Å (20,000× nominal magnification) or 6.14 Å (10,000× nominal magnification) on the level of the specimen. Bidirectional tilt series were semi-automatically collected with 2° angular increments using the SerialEM package^53^. A cumulative electron dose between 120 and 140 e^−^ Å^−2^ was used. All frames were motion corrected and damage compensated using python scripts (DE_process_frames-2.8.1.py and DE_damage_compensate-1.0.0.py) provided by Direct Electron. Tomograms were reconstructed from the aligned images and CTF corrected by phase flipping using the IMOD software package^54, 55^. 3D volumes were segmented manually using the Amira software program (FEI Visualization Sciences Group, Hillsboro, OR).

Linear density profiles were measured from the tomographic slices in Fiji software^28, 56^. The number of glycoproteins and viral membrane lengths were measured from the tomographic data; model points (scattered points) were placed on the glycoproteins and the viral membrane (open contours) and the IMOD command imodinfo was used to extract the quantitative data. Power spectra were generated in IMOD using the FFT tool. Graphs and statistical analyses were done with GraphPad Prism version 6.0.

RNP length quantification was carried out using the reconstructed tomographic data in IMOD package, similarly to what has been published^27, 28^. Briefly, model points were placed as open contours along the RNP in the tomogram (Supplementary Figure 10, Supplementary Movies 9–10). M-coated and uncoated RNP length measurements were done separately. RNP segment lengths were extracted using the imodinfo command and were added together to represent the total RNP length in the released MeV particle.

The H glycoprotein organization was analyzed using either the tomographic slices or the projections onto the *XY* plane along the *Z*-axis. For the analysis, the recMeV-(H-118∇41×) strain was used since the H and F glycoproteins can be distinguished based on their height differences (Supplementary Figure 2). Power spectra of the single tomographic slices at the assembly sites were generated using the EMAN2 command^57^ e2proc2d.py input.mrc output_powerspectrum.mrc --process*=*math.realtofft. Volumes of the F and H layer (~8 nm thickness) were projected onto the *XY* plane along the *Z*-axis using the IMOD command xyzproj, separately. The power spectra of the 2D projections were generated using EMAN2 command as described above (Supplementary Figure 7). The threshold was adjusted manually to a level that the ordering of the glycoproteins could be identified by the reflections.

### Sub-volume averaging

Sub-volume averaging was performed using the PEET software package^40, 58^. In some cases when the center of the mask was different from the center of the sub-volume, binary masks were generated with SPIDER^59^. All tomograms used for sub-volume averaging were collected at a pixel size of 2.94 Å, then binned by a factor of 2. Tomographic volumes were normalized using e2proc3d.py command (EMAN2)^60^ prior to iterative alignment and refinement in PEET (version 1.11.0 alpha). The parameters used for each structure are summarized in Supplementary Table 3.

### Sub-volume averaging of the M lattice

For both Edm and recMeV-(H-118∇41×) strains, the sub-volumes were manually picked from IMOD Slicer Window and centered on the M layer from low-pass filtered tomograms (binned by 4). The distance between the nearest sub-volumes was around 12 pixels (~ 7 nm). The initial orientations of the sub-volumes were determined by rotating the tomogram so that the M lattice was parallel to the *XY* plane and the RNP was vertical in the *XY* view in the IMOD Slicer Window. An initial reference model was generated from the sub-volumes with the pre-determined rotations and averaged in PEET. Sub-volume averages were iteratively generated and refined using the tomographic data binned by 4 (pixel size 11.76 Å), and then the coordinates scaled by 2 were applied to the tomographic data binned by 2 (pixel size 5.88 Å). Sub-volume dimensions (*X*, *Y*, *Z*) in pixels (binned by a factor of 2) were 80, 80, and 64. A soft-edge cylindrical mask (radius of 32 pixels and height of 16 pixels at binning by 2) was applied to the M layer. The height of the mask was set to exclude the densities corresponding to the glycoproteins and the RNP complex. The PEET command modifyMotiveList and the initial *XYZ* angles from the Slicer Window were used to create the initial motive lists. To compensate for the effect of the missing wedge on the tomographic data, 8 weighted groups were used during the alignment and averaging process in PEET. To avoid density bias between sub-volumes, individual sub-volumes were normalized. The initial reference model was generated from the unaligned sub-volumes with the pre-determined rotations from IMOD Slicer window and averaged in PEET. Iterations of alignment in 6 dimensions (translations and rotations) were performed with decreasing search distances and angles; the reference was refined at each iteration step. Duplicate and low cross-correlation coefficient (CCC) valued sub-volumes were removed after iterative alignment and refinement. Sub-volumes within a distance of 10 pixels (binned by 2) were treated as duplicates and all sub-volumes but those with the highest CCC were removed (~50% were removed (1437 out of 2540 were removed for recMeV-(H-118∇41×); 1241 out of 2200 were removed for Edm MeV). The CCC value was set to the average CCC of all the remaining sub-volumes and another ~50% were removed (478 out of 1103 were removed for recMeV-(H-118∇41×); 473 out of 959 were removed for Edm MeV). After removing the duplicates and low-CCC valued sub-volumes, ~25% (625 out of 2540 for recMeV-(H-118∇41×); 486 out of 2200 for Edm MeV) of the total sub-volumes were averaged into the final reconstruction^61^. No symmetry was applied during the alignment and averaging process. The averaged structure was low-pass filtered to 35 Å according to the frequency at FSC 0.5 cutoff (Supplementary Table 3, Supplementary Figure 12).

### Sub-volume averaging of the F lattice

Sub-volume averaging of the F lattice was done in a similar way as the M lattice, except that the sub-volumes (96 × 96 × 96 pixels) were picked and centered on the F layer from the recMeV-(H-118∇41×) tomographic data and a soft-edge cylindrical mask (radius of 32 pixels and height of 72 pixels) was used to include only the F layer. Iterative alignment was done similarly to the M lattice. Briefly, the initial reference model was generated from the sub-volumes with the pre-determined rotations from IMOD Slicer Window and averaged in PEET. Iterations of alignment in 6 dimensions (translations and rotations) were performed with decreasing search distances and angles, and the maximum search distance in *X* and *Y* directions was 16 pixels. Missing wedge compensation was enabled with 8 weighted groups and the reference was refined at each iteration step. No symmetry was applied during the alignment and averaging process. The averaged structure was low-pass filtered to 45 Å according to the frequency at FSC 0.5 cutoff (Supplementary Table 3, Supplementary Figure 12).

### Sub-volume averaging of the F glycoprotein trimer

The sub-volume alignment process was similar to the protocol we used for the RSV F glycoprotein averaging^34^. Sub-volumes (64 × 64 × 64, 37.6 nm) were extracted from the reconstructed tomograms. The distance between the nearest sub-volumes was 16 pixels (9.4 nm). SpikeInit (PEET command) was used to determine the initial Euler angles and these initial Euler angles were used to generate an initial model of F. A cylindrical mask (radius of 12 pixels and height of 28 pixels) was used to eliminate the neighboring densities. We generated an averaged structure of MeV F from a relatively homogeneous F only region on a recMeV-(H-118∇41×) virus particle using top and side views, taking advantage of the height differences between glycoproteins H and F (Supplementary Figure 2). Since the MeV F glycoprotein is known to be a trimer and can be seen in the tomogram as such, we applied three-fold (C3) symmetry by rotating the sub-volumes 120 degrees and 240 degrees around the *Y* axis in the motive list file. Iterative alignment and averaging steps were performed in 6 dimensions (translation and rotation), with decreasing search distances and angles at each iteration step; the reference was refined after each iteration step. In order to generate the averaged F structure from the mixed population of F and H in recMeV-(H-118∇41×) sample, we extracted sub-volumes of F from the top and side views based on the glycoprotein height differences in H and F (Supplementary Figures 2, 4). The averaged F structure from above was used as the initial reference, and the process was done similarly to the methods described above.

### Sub-volume averaging of the F–M lattice

Sub-volume averaging of the F-M lattice in both Edm and recMeV-(H-118∇41×) data sets was done similarly to the methods described above for the M lattice. Briefly, sub-volumes (80, 80, 128 pixels, binned by 2) were picked and centered on the M layer and a soft-edge cylindrical mask (radius of 32 pixels and height of 72 pixels) was used to exclude regions outside of the F and M layers. After iterative alignment and averaging at the binning by 4 level, duplicate sub-volumes and low-CCC valued sub-volumes were removed prior to final alignment and averaging at the binning by 2 level. The procedures and criteria for removing the duplicate and low-CCC valued sub-volumes for F-M lattice is the same as the M lattice. Briefly, about ~50% (1357 out of 2540 were removed for recMeV-(H-118∇41×); 1138 out of 2200 were removed for Edm MeV) of duplicate sub-volumes were removed, and another ~50% (574 out of 1183 were removed for recMeV-(H-118∇41×); 598 out of 1062 were removed for Edm MeV) of the low-CCC valued sub-volumes were removed from the remaining sub-volumes^61^. About 25% (609 out of 2540 for recMeV-(H-118∇41×); 464 out of 2200 for Edm MeV) of the particles were used for final reconstruction (Supplementary Table 3). References were refined after each iteration and missing wedge compensation was enabled during the alignment and averaging process.

### Sub-volume averaging of the RNP helix

Sub-volume averaging of the RNP helical structure was done in PEET. The sub-volumes were picked in the IMOD Slicer Window by rotating the RNP to be vertical (going down the *Y* axis). The *XYZ* angles from the Slicer Window were used to generate the initial motive lists using the command modifyMotiveList. The initial model of the RNP helix was created using a small set of sub-volumes, and the initial helix pitch (85.6 Å) was determined by measuring the distance to make a full helix turn from the initial model. We applied helical symmetry based on the known parameters of the MeV RNP structure^10, 11, 22, 48, 49^, a one-start left-handed helix with ~13 subunits per turn, by modifying the rotational and translational parameters of each sub-volume in the csv files (the aligned particle coordinates from PEET) using the modifyMotiveList command. For alignment, a cylindrical mask (64 pixels in height) was used with an inner radius of 4 pixels and an outer radius of 24 pixels (pixel size 5.88 Å), and missing wedge compensation was enabled. After iterative refinement and averaging, the global averaged structure showed a clear left-handed helix, with stronger density in the central segment of the helix while the two ends were weaker. We performed classification using principle component analysis (PCA) and K-means clustering with a cylindrical mask and generated class averages. When running K-means clustering after PCA with the command clusterPca, we used 2, 4, 6, or 8 as the nCluster parameter. The best results were obtained using 2 classes (1841 sub-volumes in class 1 and 1084 sub-volumes class 2). When using greater than 2 as the nCluster parameter, classes were generated based on the missing wedge artifact instead of the variance in the RNP structure. Two classes of the RNP were generated as shown in Fig. 5. The PEET/IMOD commands createAlignedModel and clonemodel were used to place the sub-volume averaged structure back into the raw tomogram. The EM density of the RNP helix was visualized in Chimera software^62^.

### Variance map and the organization of H glycoprotein

We did not detect ordering of the H glycoprotein as seen in the M and F 2D lattices (seen in tomographic data, power spectra, sub-volume averages in Supplementary Figure 7). Tomograms of recMeV-(H-118∇41×) virus did not contain ordered arrays of H, neither did the power spectra of tomographic slices or sub-volume averages (Supplementary Figure 7). In order to assess the lack of ordering of the H glycoprotein, we generated variance maps from sub-volumes used to generate the initial model and the aligned class average (Supplementary Figure 4). To calculate the variance, we applied a mask including only the F–H region, with the averaged structure being the trueFile (PEET input, MRC format file representing an estimation of the true particle). All particles, except for the duplicates and low-CCC valued sub-volumes, and the final iteration number were used in the varianceMap analysis. The highest variance was seen in the H layer and not the F layer or M layer (Supplementary Figure 5).

### Model fitting

X-ray crystal structures of the MeV M and F homologs were used for model fitting in Chimera software^62^. The dimeric crystal structure of NDV M (PDB ID: 4GIL) was manually fitted into the low-pass filtered M lattice EM density map as rigid body^16^. The trimeric crystal structure of PIV5 prefusion F (PDB ID: 2B9B, with the trimeric coiled-coil GCNt domain removed)^37^ was manually fitted as rigid body into the low-pass filtered F glycoprotein EM density. Visualization and image rendering were done using Chimera program^62^.

### Data availability

All relevant data are available from the corresponding author upon reasonable request. All sub-volume averages have been deposited in the Electron Microscopy Data Bank (www.emdatabank.org) under the following accession numbers: EMD-7565, EMD-7566, EMD-7587, EMD-7588, EMD-7590, EMD-7591, EMD-7594, EMD-7595, EMD-7596, and EMD-7597. Refer to Supplementary Table 3 for details.

## Electronic supplementary material

Supplementary Information

Description of Additional Supplementary Files

Supplementary Movie 1

Supplementary Movie 2

Supplementary Movie 3

Supplementary Movie 4

Supplementary Movie 5

Supplementary Movie 6

Supplementary Movie 7

Supplementary Movie 8

Supplementary Movie 9

Supplementary Movie 10
