# Supplementary material for: Promotion of virus assembly and organization by the measles virus matrix protein
Source: Nat Commun. 2018 Apr 30;9:1736. doi: 10.1038/s41467-018-04058-2 (PMC5928126; doi:10.1038/s41467-018-04058-2)
Supplement: Supplementary file 1 — Supplementary Information [file 41467_2018_4058_MOESM1_ESM.pdf]

# Promotion of Virus Assembly and Organization by the Measles Virus Matrix Protein

Ke *et al.*

**SUPPLEMENTARY INFORMATION.**

**LEGENDS FOR SUPPLEMENTARY FIGURES, SUPPLEMENTARY TABLES, AND SUPPLEMENTARY REFERENCES.**

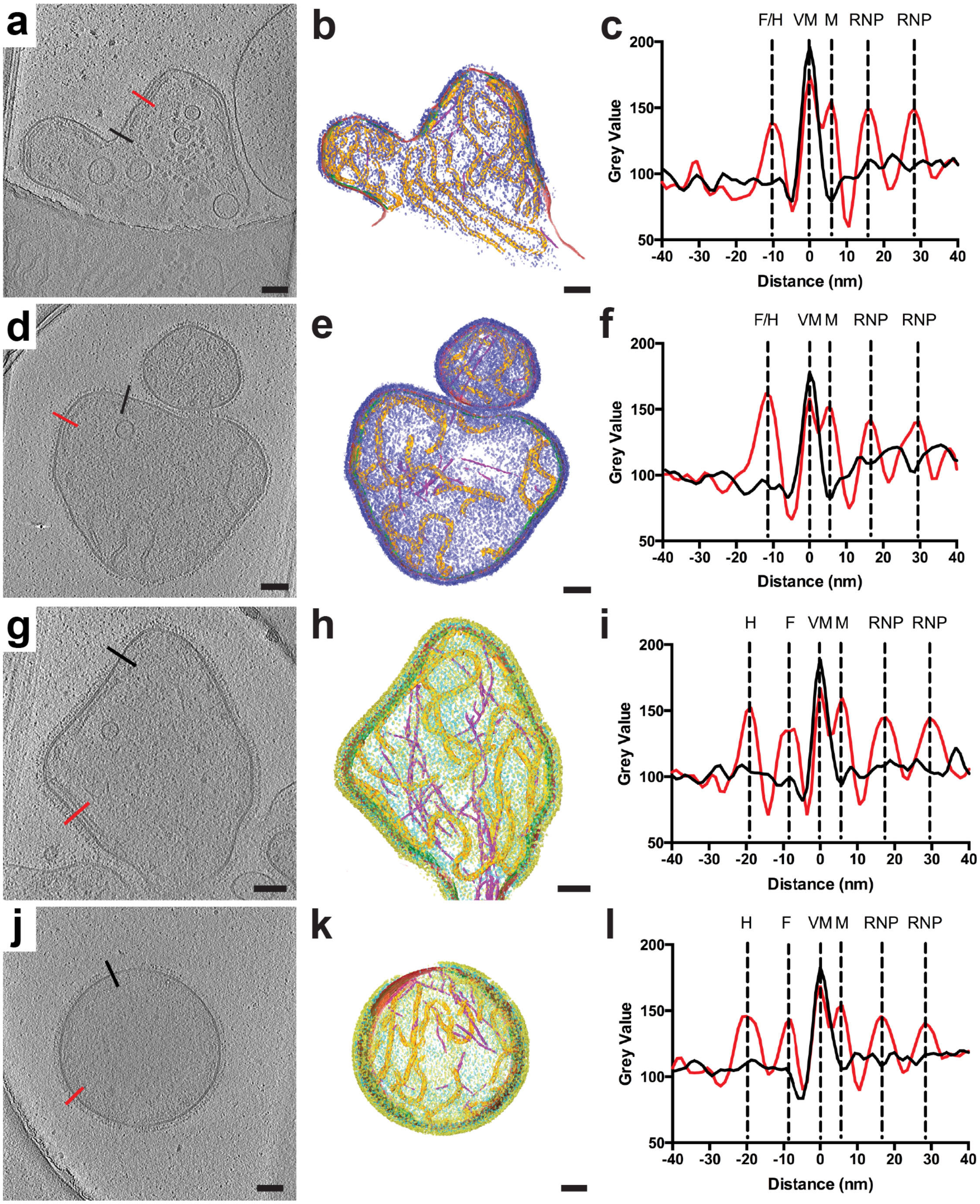
a
b
c
F/H
VM
M
RNP
RNP
Grey Value
Distance (nm)
d
e
f
F/H
VM
M
RNP
RNP
Grey Value
Distance (nm)
g
h
i
H
F
VM
M
RNP
RNP
Grey Value
Distance (nm)
j
k
l
H
F
VM
M
RNP
RNP
Grey Value
Distance (nm)

**Supplementary Figure 1 | Cryo-ET of MeV-infected cells indicates matrix protein drives organization of glycoproteins and RNP in Edm and recMeV-(H-118∇41×).**

Tomographic slices of Edm MeV (a, d) and recMeV-(H-118∇41×) (g, j) at the assembly site (a, g) and released MeV particles (d, j). The M protein does not cover the entire viral membrane and gaps were observed as indicated by the black lines. Corresponding segmented views of Edm (b, e) and recMeV-(H-118∇41×) (h, k). (b, e, h, k): viral membrane (VM) is red, M is green, glycoproteins are blue (Edm) or cyan (F in recMeV-(H-118∇41×)) and yellow (H), RNPs are orange, and actin is magenta. Linear density profiles through MeV assembly site (c, i) and released MeV (f, l). Density peaks of the different viral components are indicated by the dashed lines, including the glycoproteins (F/H), viral membrane (VM), M protein (M) and the RNPs. Scale bars are 100 nm.

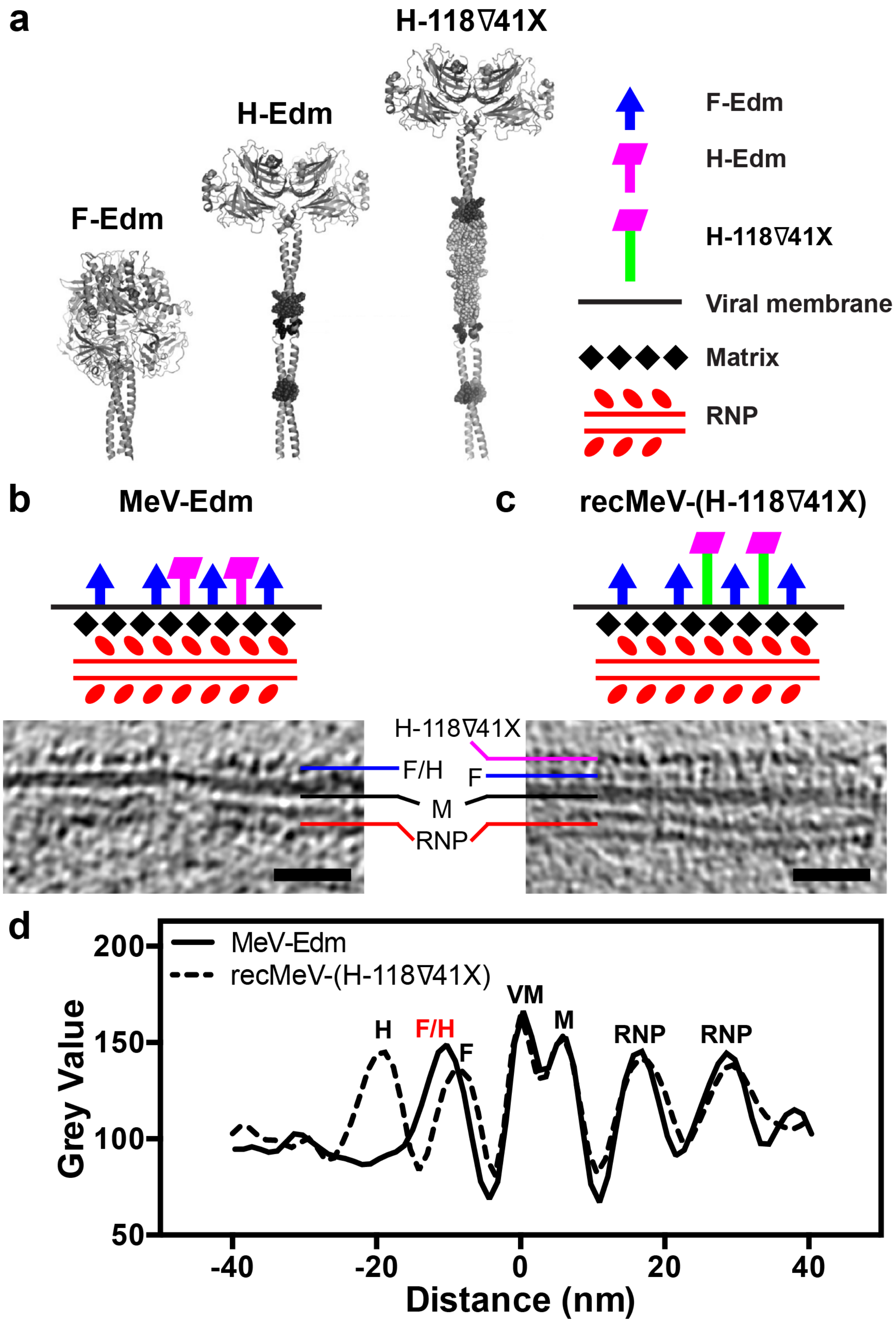
a
F-Edm
H-Edm
H-118∇41X
F-Edm
H-Edm
H-118∇41X
Viral membrane
Matrix
RNP
b
MeV-Edm
c
recMeV-(H-118∇41X)
H-118∇41X
F/H
F
M
RNP
d
MeV-Edm
recMeV-(H-118∇41X)
H
F/H
F
VM
M
RNP
RNP
Grey Value
200
150
100
50
-40
-20
0
20
40
Distance (nm)

**Supplementary Figure 2 | MeV glycoprotein differences between the MeV-Edm and recMeV-(H-118∇41×) strains.** (a) Model of MeV glycoproteins on the left and cartoon model of MeV structural components on the right. The structural model from panel a was adapted from Paal *et al.*, 2009[1]. (b and c) Cartoon representation (top) and tomographic slices (bottom) of the two strains investigated in this study. Note the clear difference in the height of F and H in recMeV-(H-118∇41×) strain. Scale bars are 50 nm. (d) Linear profiles of the two strains. The MeV-Edm strain has only one glycoprotein (F/H) peak while the in recMeV-(H-118∇41×) strain has two separate peaks for F and H.

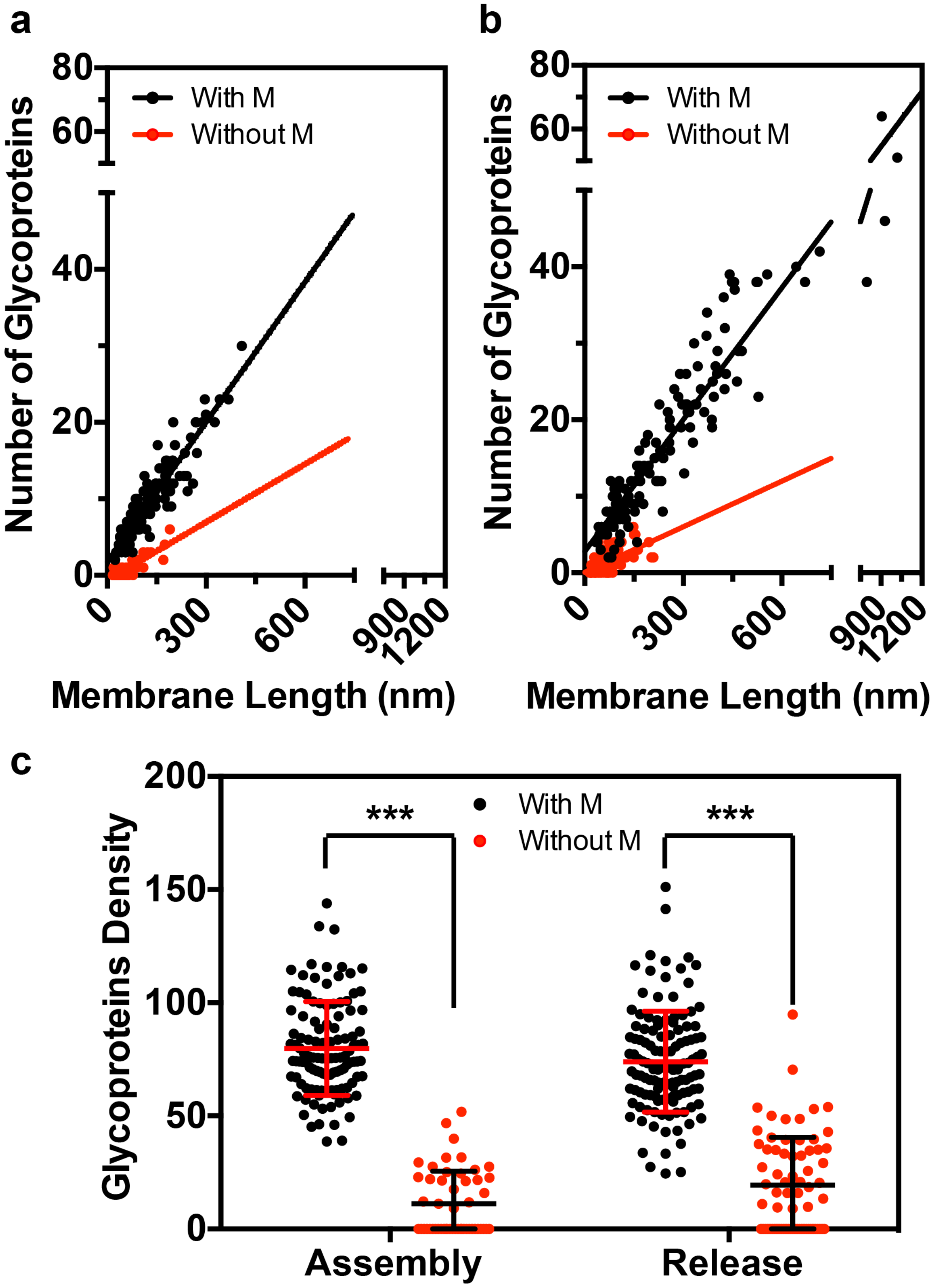


**Supplementary Figure 3 | M protein regulates incorporation of glycoproteins into the viral membrane at sites of assembly and in released MeV.** (a-b) The number of

glycoproteins incorporated into the viral membrane of Edm MeV with M present (black dots) and areas of the viral membrane that lack M (red dots) at the assembly sites (a) and in the released virus particles (b). (c) Glycoprotein density of MeV at the assembly sites and in the released virus particles in the presence and absence of M. Note, most of the membrane has M underneath and the presence of M is associated with longer membrane lengths, indicated by black dots (a and b). The error bars represent the s.d. of 113 (assembly with M), 50 (assembly without M), 126 (released virus with M), and 70 (released virus without M) measurements indicated by the graph data points, the data were analyzed using *t*-test. When significant, p values are shown as a bracket between groups by asterisks. *** indicates $p < 0.001$.

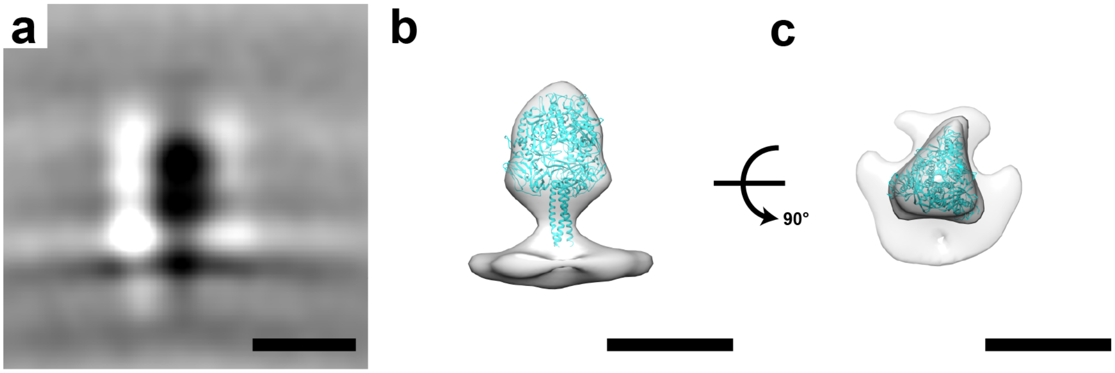


**Supplementary Figure 4 | Sub-volume average of MeV F glycoprotein from a mixed population of H and F glycoproteins.** (a) Central slice of the averaged F structure. (b-c) Side and top views of the averaged F glycoprotein trimer, validated by model fitting of PIV5 pre-fusion F structure (PDB ID: 2B9B). Scale bars are 10 nm.

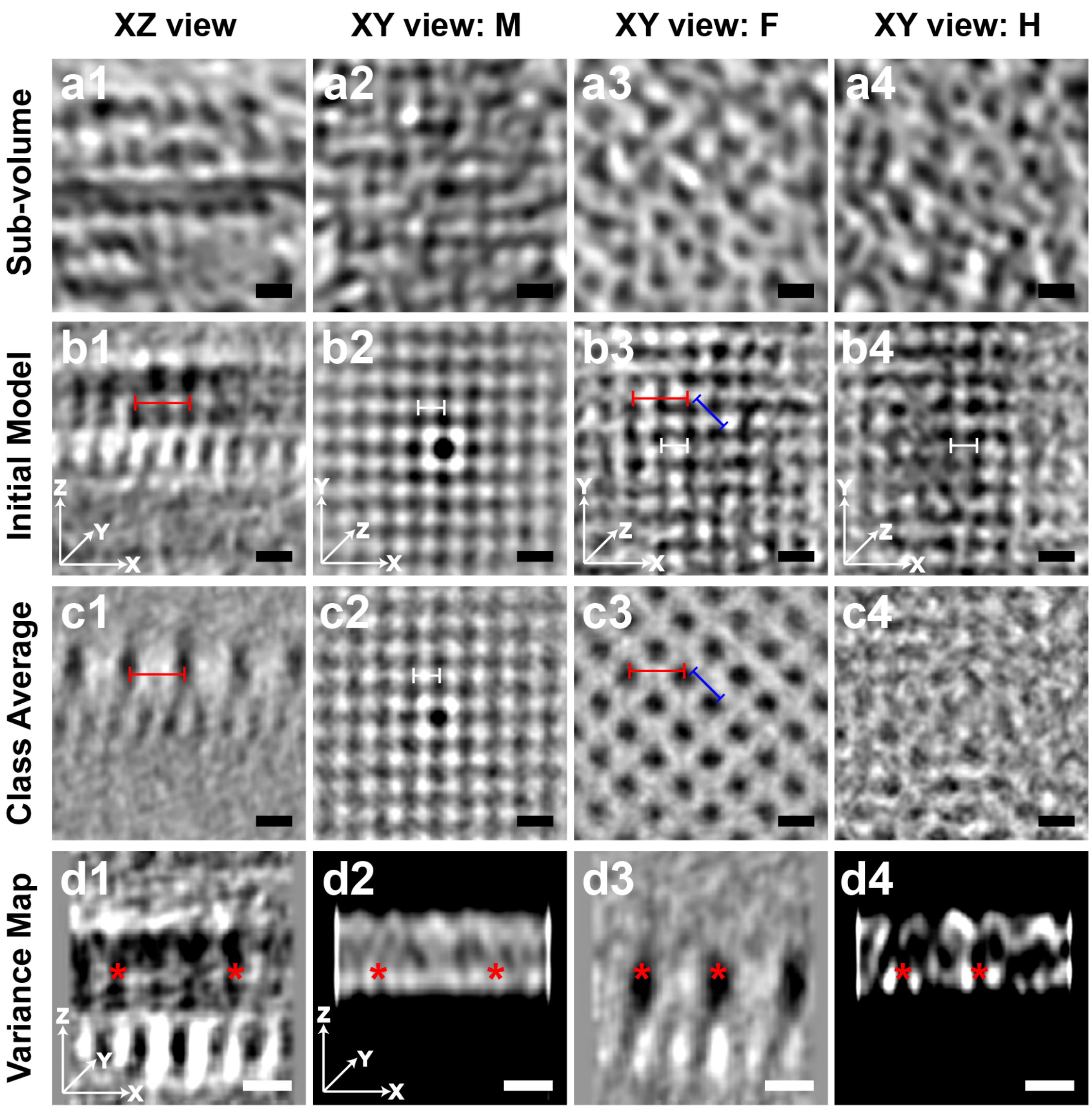


**Supplementary Figure 5 | H glycoprotein does not form a 2D lattice and is highly variable.** (a1-a4) Representative views of initial sub-volumes extracted from raw tomograms of the recMeV-(H-118∇41×) strain. Note, due to missing wedge in the tomogram, the slices were from the same tomogram but not the same location. (b1-b4) Tomographic slices of initial model, indicating there are densities at the H layer, along with M lattice and F lattice. (c1-c4) Classification results indicate that the lattice of M and F preserves, while H layer density is very weak and loss of regularity. (d1-d4) Variance

maps of initial model (d1-d2) and class average (d3-d4). d1 and d2 indicate that there is high level of variance in the glycoproteins, most likely due to mixture population of H. Asterisks in d1 (initial model b1) correlate with asterisks in d2 (variance). Asterisks in d3 (class average c1) correlate with asterisks in d4 (variance). Black is density in the density maps (a1-c4, d1, d3) while black is low variance and white is high variance in variance maps (d2, d4). The red lines (true spacing of diagonal F) indicate 15.6 nm, white lines indicate 7.8 nm (true spacing of M, artifact spacing of F and H layers), and the blue lines (true spacing of the nearest F) indicate 11 nm. Scale bars are 10 nm.

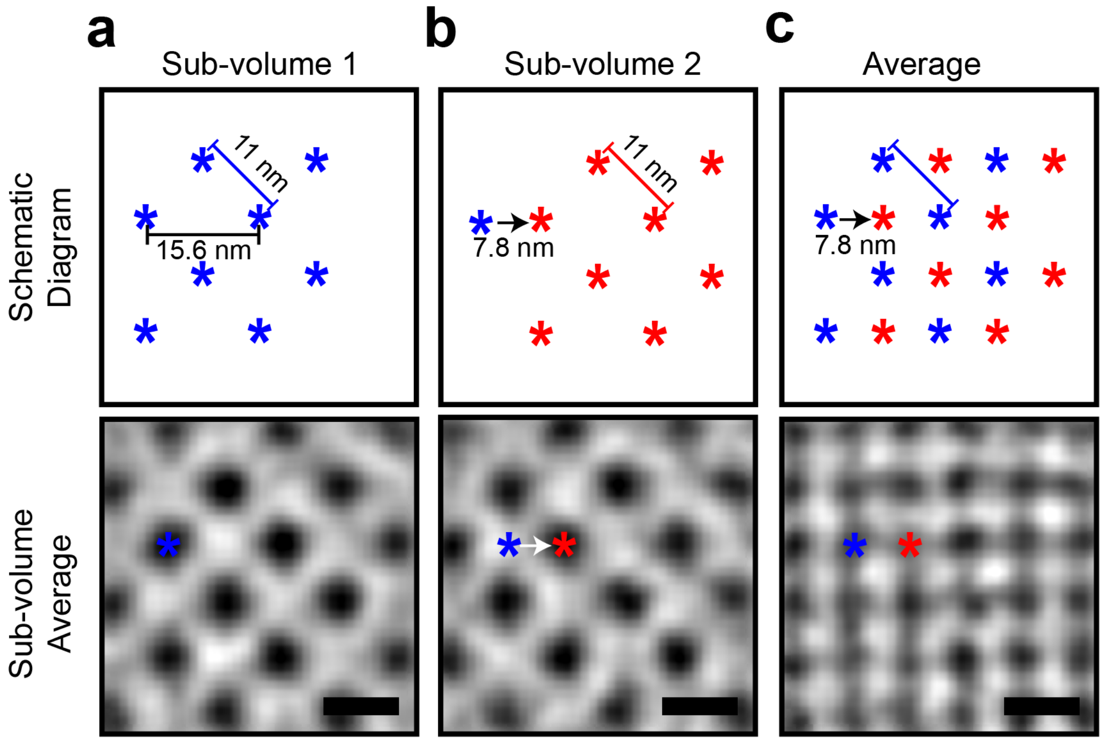


**Supplementary Figure 6 | The organization of F lattice during the sub-volume averaging process.** (a) The spacing of the F glycoprotein lattice on XY view, sub-volume 1. The spacing of F lattice is 11 nm. (b) Shift the whole sub-volume 1 to the right by the spacing of M lattice (7.8 nm) to get sub-volume 2. F lattice shifts from the blue (a) to the red (b). (c) The average of the two sub-volumes (1 and 2) without alignment results a mis-represented average (spacing of 7.8 nm) of the F lattice while M lattice will still be perfectly aligned with a spacing of 7.8 nm. This helps explaining the spacing in **Supplementary Figure 5**. Top panels are the schematic diagrams, bottom panels are sub-volume average slices. The blue asterisks represent F subunit density in sub-volume 1, the red asterisks represent F subunit density in sub-volume 2, and the white arrow indicates the shift spacing of 7.8 nm. Scale bars are 10 nm.

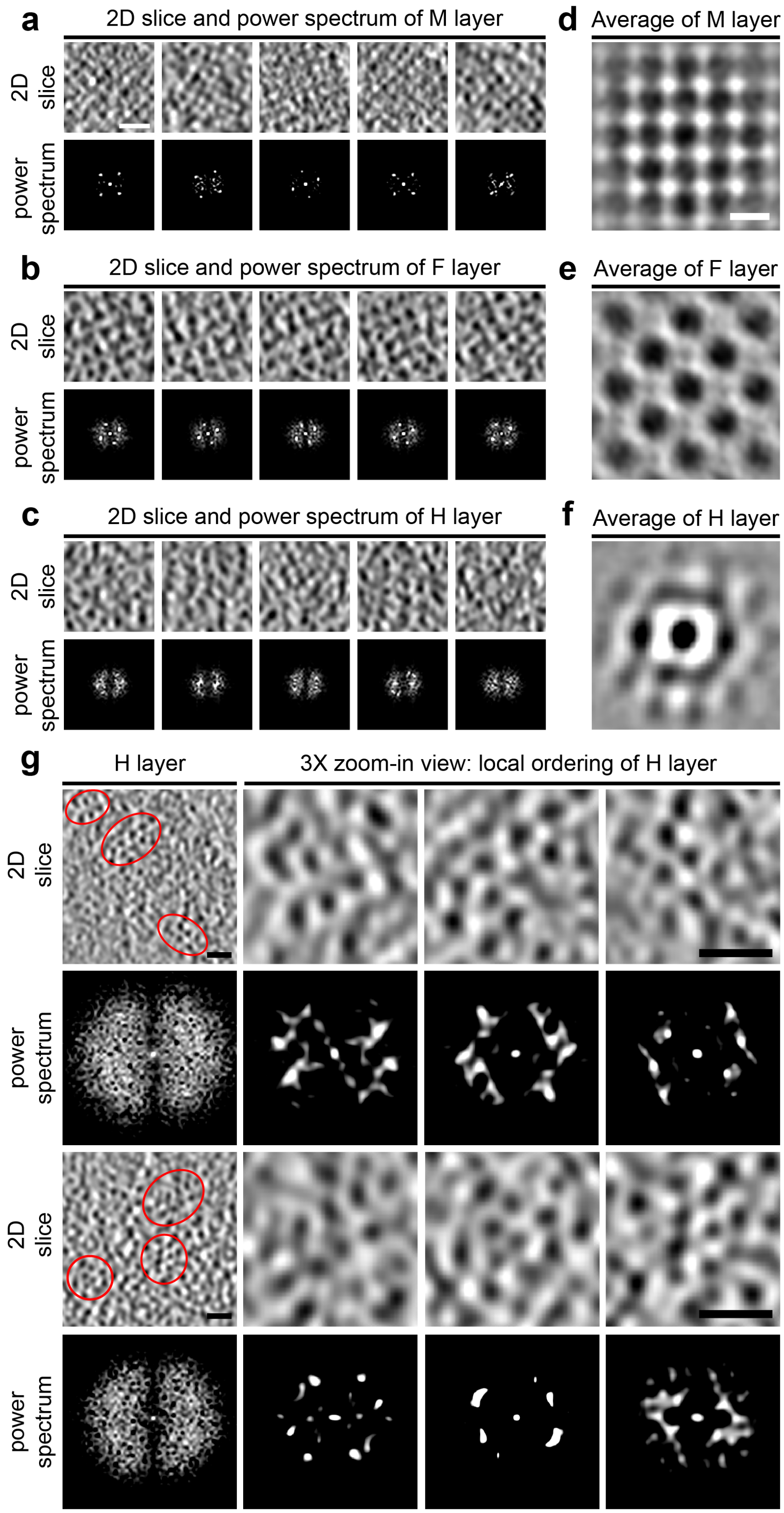
a
2D slice and power spectrum of M layer
2D slice
power spectrum
d
Average of M layer
b
2D slice and power spectrum of F layer
2D slice
power spectrum
e
Average of F layer
c
2D slice and power spectrum of H layer
2D slice
power spectrum
f
Average of H layer
g
H layer
3X zoom-in view: local ordering of H layer
2D slice
power spectrum
2D slice
power spectrum

**Supplementary Figure 7 | The ordering of MeV membrane-associated proteins from recMeV-(H-118∇41×) strain.** (a) 2D slice and power spectrum of the M layer. The top panels are the representative 2D slices from different volumes, and the bottom panels are the corresponding the power spectra. Note there is ordering on M layer. (b) 2D slice and power spectrum of the F layer. The top panels are the representative 2D slices from different volumes, and the bottom panels are the corresponding the power spectra. Note there is ordering on F layer. (c) 2D slice and power spectrum of the H layer. The top panels are the representative 2D slices from different volumes, and the bottom panels are the corresponding the power spectra. Note there is no ordering on H layer. (d-f) Sub-volume averaging of the M, F, and H layers, separately. (g) Examples of the local ordering of H layer indicated by the red circles and corresponding 3X zoom-in views. Scale bars are 20 nm (a and g) and 10 nm (d).

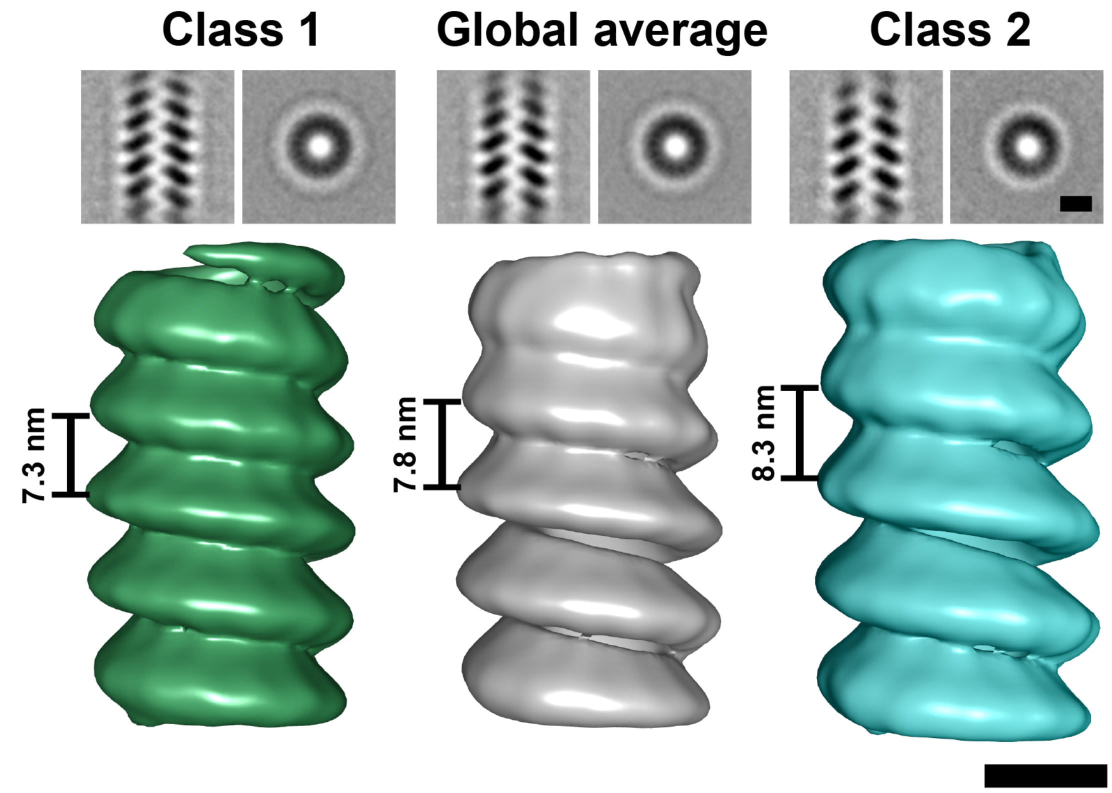


**Supplementary Figure 8 | MeV RNP classifications.** (Top) Tomographic slices of the top and side views of each class average and global average. (Bottom) Isosurface rendering of each sub-volume average. The colour scheme is the same as **Figure 5**. Black is density. Scale bars are 10 nm.

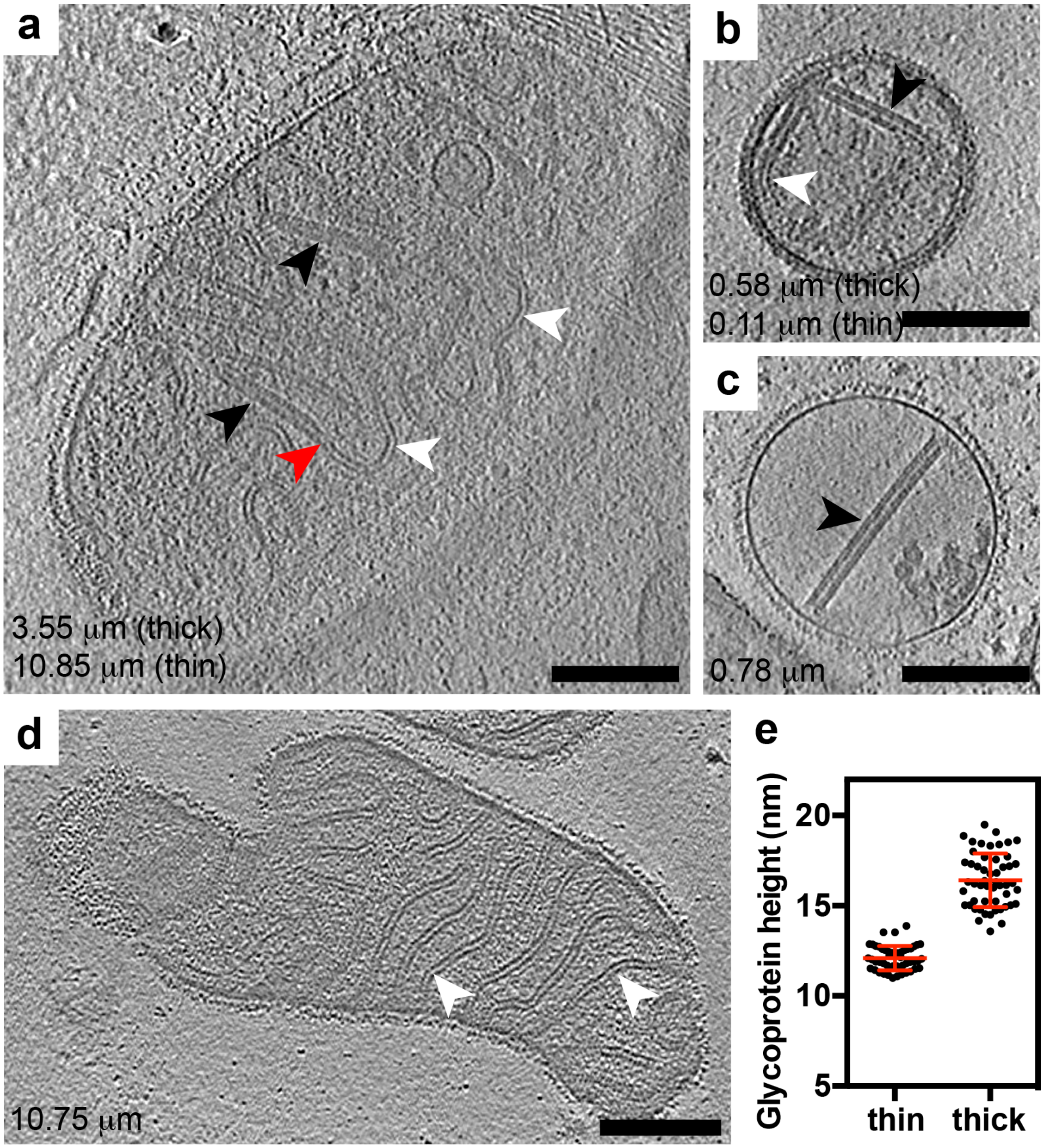


**Supplementary Figure 9 | Coated and uncoated RNP from MeV-infected cells.** (a-c) Tomographic slices of M-coated RNP (thick) examples. White arrowheads indicate uncoated RNP (thin) and black arrowheads indicate M-coated RNP (thick). The red arrowhead indicates the connection between thick and thin RNP from a continuous linear RNP. (d) An example of a released MeV with uncoated RNP. Numbers (a-d) on the bottom left indicate the RNP length. (e) Glycoprotein height measurements indicate

the glycoproteins are shorter (12.1 nm, likely pre-fusion state) when RNP is uncoated (thin) than the glycoproteins (16.4 nm, likely post-fusion state) when RNP is coated (thick). The error bars represent the s.d. of 50 measurements (thin) and 52 measurements (thick) indicated by the graph data points. Scale bars are 200 nm.

a

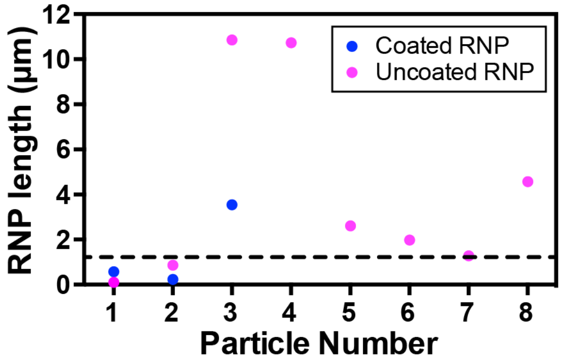


b

| Particle # | RNP length / µm | |
|---|---|---|
| | Coated RNP | Uncoated RNP |
| 1 | 0.58 | 0.11 |
| 2 | 0.24 | 0.86 |
| 3 | 3.55 | 10.85 |
| 4 | NA | 10.75 |
| 5 | NA | 2.63 |
| 6 | NA | 2.01 |
| 7 | NA | 1.31 |
| 8 | NA | 4.60 |

c

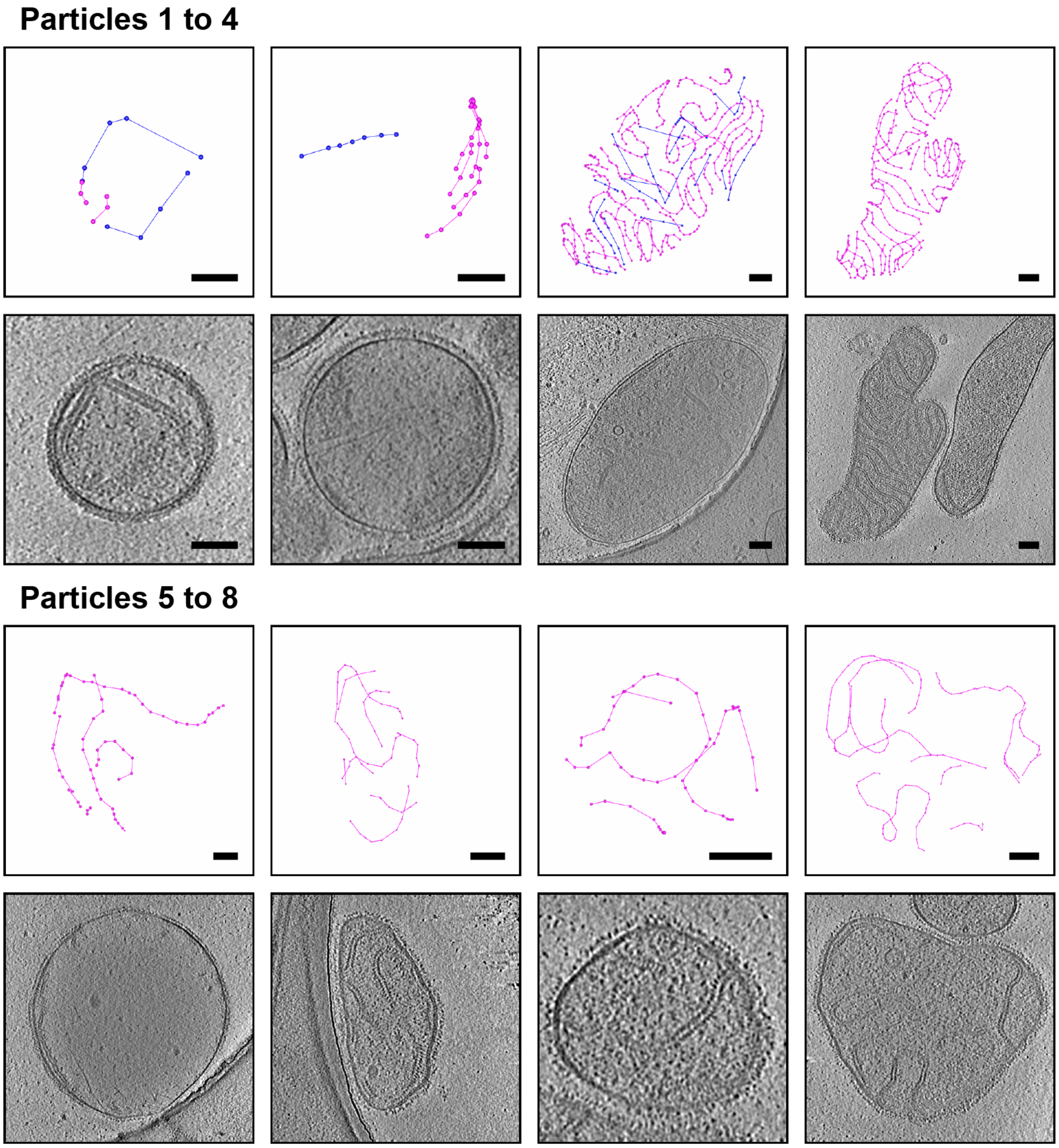

**Supplementary Figure 10 | Quantification of RNP length from Edm MeV particles.** (a) Individual RNP length measurement from tomographic reconstruction. The dashed line indicates one genomic copy (1.3 µm) of the RNP length. (b) A table shows the measurements plotted in a. (c) Model views of the RNP length (top panels) and corresponding tomographic slices (bottom panels) of the 8 MeV particles. Blue lines indicate coated RNP and the pink lines indicate uncoated RNP. Particles 1, 3, and 4 are shown in **Supplementary Figure 9**. Scale bars are 100 nm.

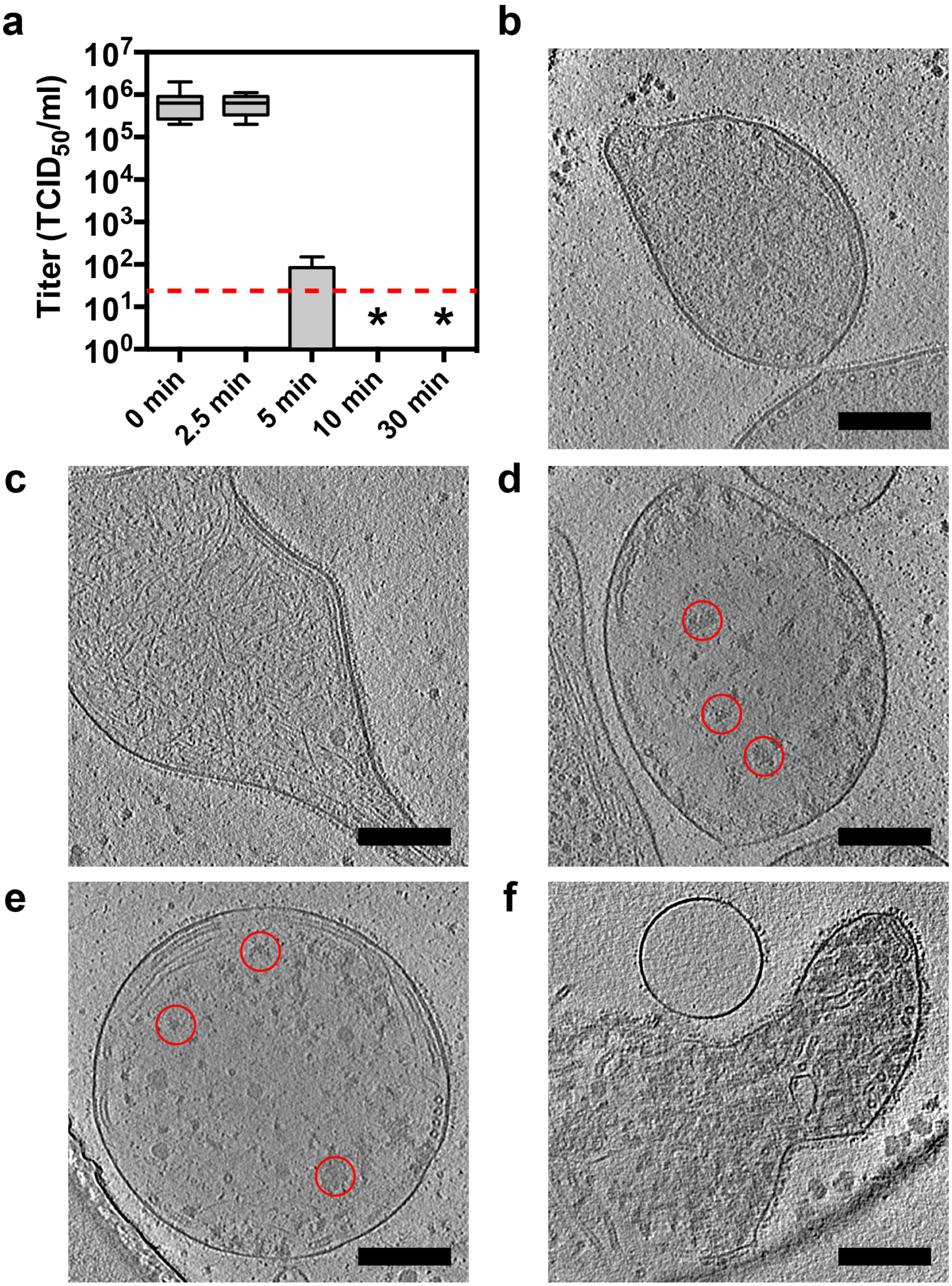


**Supplementary Figure 11 | Heat-treatment affects MeV structural proteins organization.** (a) Titration of the MeV-infected HeLa cells over 30 minutes time period

when heat-treated at 60 ºC. Limit of detection is indicated by the red dashed line. * indicates the undetected titer at 10 and 30 min time points. The error bars represent the s.d. of three independent experiments. The box plot indicates the upper (3rd quartile) and lower limit (1st quartile) of the range. (b-f) Representative tomographic slices of MeV particles at designated time points, i.e., 0 min (b), 2.5 min (c), 5 min (d), 10 min (e), and 30 min (f), corresponding to the titration experiment. Note the detachment of the M layer at time points 5 min and longer. Broken viral particles were frequently observed at 30-min time point. The red circles indicate the aggregated proteins inside the heated particles (d and e). Scale bars are 200 nm (b-f).

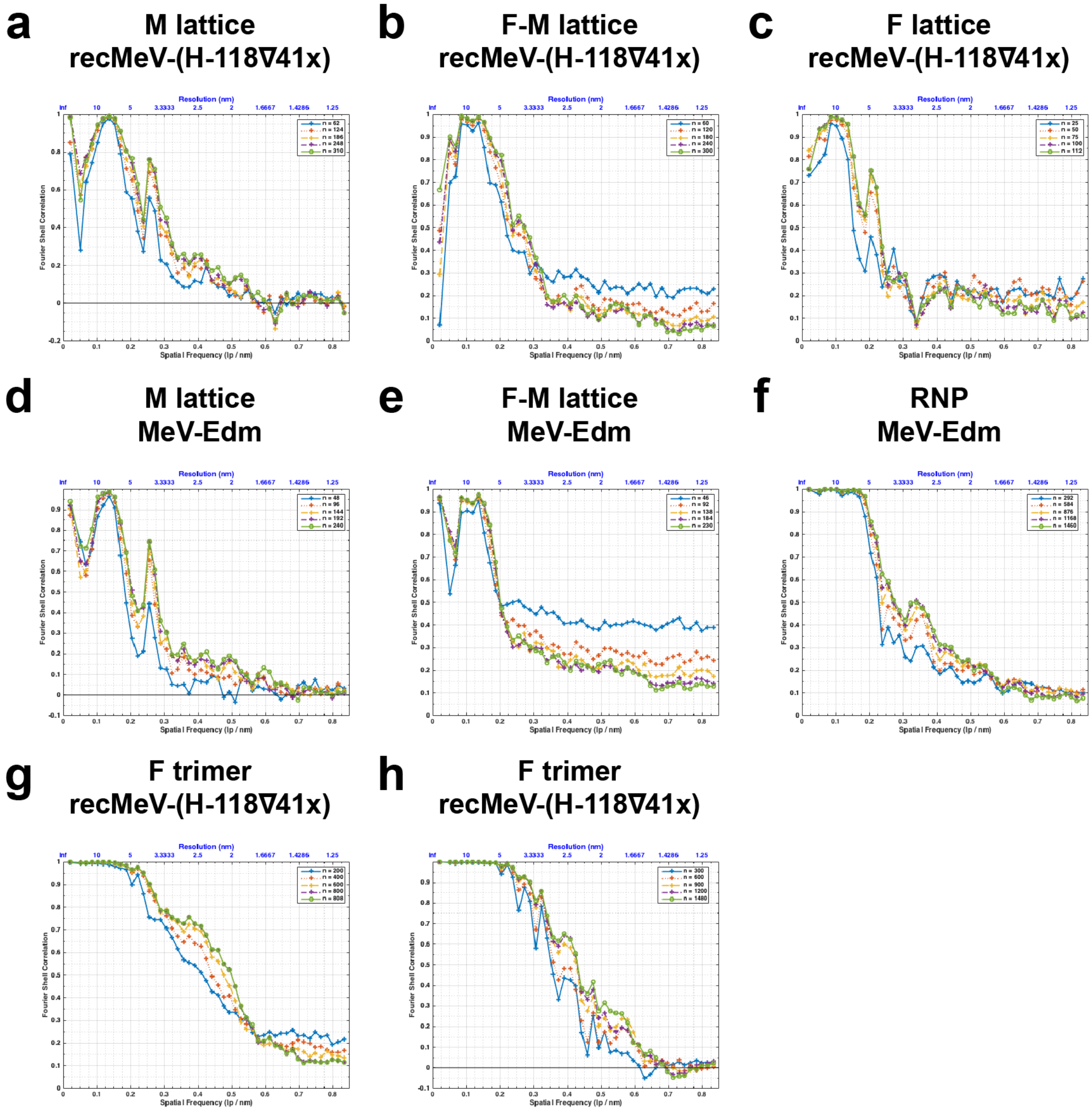


**Supplementary Figure 12 | FSC curves of the sub-volume averages in this study.**

The particle numbers used are indicated in the insets. The pixel size of the final reconstructions is 5.88 Å. This figure corresponds to **Supplementary Table 3**.

**Supplementary Table 1 | Tomograms of MeV assembly sites and released MeV particles from infected HeLa and MRC-5 cells.**

| Cell Line | MeV Strain | Tomograms | Assembly | Budding | Released | Total |
|---|---|---|---|---|---|---|
| HeLa | Edm | 20 | 12 | 11 | 9 | 32 |
| HeLa | recMeV-(H-118∇41×) | 20 | 17 | 8 | 13 | 38 |
| MRC-5 | Edm | 10 | 4 | 2 | 4 | 10 |
| MRC-5 | recMeV-(H-118∇41×) | 7 | 3 | 3 | 3 | 9 |

**Supplementary Table 2 | Cryo-ET data collection parameters.**

| | |
|---|---|
| Microscope | JEOL JEM-2200FS (FEG, Omega energy filter) |
| Voltage (kV) | 200 |
| Camera | DE-20 |
| Frame rate (fps) | 24 |
| Magnification (x) | 20,000 |
| Pixel size (data collected) (Å) | 2.94 |
| Defocus (μm) | -4 to -8 |
| Tilt series angle coverage (°) | -64 to + 64 (Bi-directional) |
| Tilt series increment angle (°) | 2 |
| Electron dose per tomogram ($e^-$/$Å^2$) | 120 to 140 |

**Supplementary Table 3 | Sub-volume averaging statistics.**

| Macromolecular Complex | Sub-volumes | | Distance between sub-volumes** | Voxel size (X, Y, Z) | Mask Dimensions (radius, height) | Resolution at FSC 0.5 cutoff (Å) | EMDB accession number |
|---|---|---|---|---|---|---|---|
| | Initial sub-volumes | Final sub-volumes | | | | | |
| **M lattice (Edm)*** | 2200 | 486 | 12 | 80, 80, 64 | 32, 16 | 35 | EMD-7565 |
| **M lattice (rec)*** | 2540 | 625 | 12 | 80, 80, 64 | 32, 16 | 35 | EMD-7566 |
| **F-M lattice (Edm)** | 2200 | 464 | 12 | 80, 80, 128 | 32, 72 | 50 | EMD-7590 |
| **F-M lattice (rec)** | 2540 | 609 | 12 | 80, 80, 128 | 32, 72 | 40 | EMD-7591 |
| **F lattice (rec)** | 224 | 224 | 16 | 96, 96, 96 | 32, 72 | 45 | EMD-7587 |
| **F trimer (rec)** | 539 | 1617 (C3)† | 16 | 64, 64, 64 | 12, 28 | 20 | EMD-7588 |
| **F trimer (rec)** | 987 | 2961 (C3)† | 16 | 64, 64, 64 | 12, 28 | 25 | EMD-7597 |
| **RNP (Edm)** | 225 | 2925 (Helical)† | 14 | 96, 96, 96 | (4, 24)#, 64 | 35 | EMD-7594, EMD-7595, EMD-7596 |

Notes:

* Edm indicates Edm MeV strain, rec indicates recMeV-(H-118∇41×) strain.

** indicates all the pixels are binned by 2 (pixel size is 5.88 Å).

# indicates the inner and outer radii are 4 and 24 pixels for the cylindrical mask, respectively.

† indicates the symmetry applied to the final reconstruction.

## SUPPLEMENTARY REFERENCES.

1 Paal, T. *et al.* Probing the spatial organization of measles virus fusion complexes. *J. Virol.* **83**, 10480-10493, doi:10.1128/JVI.01195-09 (2009).
