# Supplementary material for: Promotion of virus assembly and organization by the measles virus matrix protein
Source: Nat Commun. 2018 Apr 30;9:1736. doi: 10.1038/s41467-018-04058-2 (PMC5928126; doi:10.1038/s41467-018-04058-2)
Supplement: Supplementary file 2 — Description of Additional Supplementary Files [file 41467_2018_4058_MOESM2_ESM.pdf]

Description of Additional Supplementary Files

File Name: Supplementary Movie 1
Description: Cryo-ET of MeV-infected HeLa cells at the assembly site. A MeV assembly site from the frozen-hydrated Edm MeV-infected HeLa cell demonstrates the viral structural organization in the context of whole-cell tomography.

File Name: Supplementary Movie 2
Description: Cryo-ET of MeV-infected MRC-5 cells at the assembly site. A MeV assembly site from the frozen-hydrated Edm MeV-infected MRC-5 cell demonstrates the viral structural organization in the context of whole-cell tomography. Scale bar is 200 nm.

File Name: Supplementary Movie 3
Description: Cryo-ET of a released MeV particle from MeV-infected cells. A released MeV particle from Edm MeV-infected HeLa cells demonstrates the viral structural organization. Note the M lattice and RNP are in register inside the native MeV particle.

File Name: Supplementary Movie 4
Description: Cryo-ET of MeV-infected cells at the assembly site. A MeV assembly site from the frozen-hydrated recMeV-(H-118∇41×) infected HeLa cell demonstrates the viral structural organization in the context of whole-cell tomography.
Note the F lattice on the surface of the native MeV particle.

File Name: Supplementary Movie 5
Description: Subvolume average of recMeV-(H-118∇41×) F-M lattice.
Tomographic slices going from the bottom to the top (round trip) of the averaged recMeV-(H-118∇41×) F-M overlaid lattice structure. Black is density in the tomographic slices. In the segmentation view, blue is F and cyan is M. Scale bar is 10 nm.

File Name: Supplementary Movie 6
Description: Subvolume average of Edm F-M lattice. Tomographic slices going from the bottom to the top of the averaged Edm F-M overlaid lattice structure.
Black is density. Scale bar is 10 nm.

File Name: Supplementary Movie 7
Description: Model of MeV structural proteins organization. Segmentation of the F-M lattice in conjunction with the RNP complex demonstrating the 3 layers of structural protein organization.

File Name: Supplementary Movie 8
Description: Clonemodel view of RNP classes within a tomogram. The subvolumes extracted from the tomogram were placed back into the original volume. The color scheme is the same as **Figure 5**. Scale bar is 100 nm.

File Name: Supplementary Movie 9
Description: RNP length quantification of a released MeV particle. Tomographic slices going from the bottom to the top (round trip) of a released MeV particle from MeV-infected cells. The color scheme is the same as **Supplementary Figure 10**. The blue lines are M-coated RNP while the pink lines are uncoated RNP. Scale bar is 200 nm.

File Name: Supplementary Movie 10
Description: RNP length quantification of a released MeV particle. Tomographic slices going from the bottom to the top (round trip) of a released MeV particle from MeV-infected cells. The color scheme is the same as **Supplementary Figure 10**. The pink lines indicate the presence of the uncoated RNP. Scale bar is 200 nm.
